# Budding Yeast ATM/ATR Control Meiotic Double-Strand Break (DSB) Levels by Down-Regulating Rec114, an Essential Component of the DSB-machinery

**DOI:** 10.1371/journal.pgen.1003545

**Published:** 2013-06-27

**Authors:** Jesús A. Carballo, Silvia Panizza, Maria Elisabetta Serrentino, Anthony L. Johnson, Marco Geymonat, Valérie Borde, Franz Klein, Rita S. Cha

**Affiliations:** 1 Department of Life Sciences, Genome Damage and Stability Centre, University of Sussex, Falmer, United Kingdom; 2 Division of Stem Cell Biology and Developmental Genetics, MRC National Institute for Medical Research, London, United Kingdom; 3 Department of Chromosome Biology, Max F. Perutz Laboratories, University of Vienna, Dr. Bohr-Gasse 1, Vienna, Austria; 4 (IMBA) Institute of Molecular Biotechnology of the Austrian Academy of Sciences, Dr. Bohr-Gasse, Vienna, Austria; 5 CNRS UMR218, Institut Curie/Centre de Recherche, UMR218, Pavillon Pasteur, Paris, France; 6 The Gurdon Institute, University of Cambridge, Cambridge, United Kingdom; National Cancer Institute, United States of America

## Abstract

An essential feature of meiosis is Spo11 catalysis of programmed DNA double strand breaks (DSBs). Evidence suggests that the number of DSBs generated per meiosis is genetically determined and that this ability to maintain a pre-determined DSB level, or “DSB homeostasis”, might be a property of the meiotic program. Here, we present direct evidence that Rec114, an evolutionarily conserved essential component of the meiotic DSB-machinery, interacts with DSB hotspot DNA, and that Tel1 and Mec1, the budding yeast ATM and ATR, respectively, down-regulate Rec114 upon meiotic DSB formation through phosphorylation. Mimicking constitutive phosphorylation reduces the interaction between Rec114 and DSB hotspot DNA, resulting in a reduction and/or delay in DSB formation. Conversely, a non-phosphorylatable *rec114* allele confers a genome-wide increase in both DSB levels and in the interaction between Rec114 and the DSB hotspot DNA. These observations strongly suggest that Tel1 and/or Mec1 phosphorylation of Rec114 following Spo11 catalysis down-regulates DSB formation by limiting the interaction between Rec114 and DSB hotspots. We also present evidence that Ndt80, a meiosis specific transcription factor, contributes to Rec114 degradation, consistent with its requirement for complete cessation of DSB formation. Loss of Rec114 foci from chromatin is associated with homolog synapsis but independent of Ndt80 or Tel1/Mec1 phosphorylation. Taken together, we present evidence for three independent ways of regulating Rec114 activity, which likely contribute to meiotic DSBs-homeostasis in maintaining genetically determined levels of breaks.

## Author Summary

Meiosis is a specialized cell division that underpins sexual reproduction. It begins with a diploid cell carrying both parental copies of each chromosome, and ends with four haploid cells, each containing only one copy. An essential feature of meiosis is meiotic recombination, during which the programmed generation of DNA double-strand-breaks (DSBs) is followed by the production of crossover(s) between two parental homologs, which facilitates their correct distribution to daughter nuclei. Failure to generate DSBs leads to errors in homolog disjunction, which produces inviable gametes. Although DSBs are essential for meiosis, each break represents a potentially lethal damage; as such, its formation must be tightly regulated. The evolutionarily conserved ATM/ATR family proteins were implicated in this control; nevertheless, the mechanism by which such control could be implemented remains elusive. Here we demonstrate that Tel1/Mec1 down-regulate meiotic DSB formation by phosphorylating Rec114, an essential component of the Spo11 complex. We also observed that Rec114 activity can be further down-regulated by its removal from chromosomes and subsequent degradation during later stages in meiosis. Evidence presented here provides an insight into the ways in which the number of meiotic DSBs might be maintained at developmentally programmed level.

## Introduction

In most sexually reproducing organisms, meiotic recombination is initiated by programmed catalysis of DNA double strand breaks (DSBs) by Spo11, an evolutionarily conserved type II topoisomerase-like transesterase [1]. In *Saccharomyces cerevisiae*, where the process is best understood, Spo11 activity requires nine additional proteins, five of which are meiosis specific (Rec102, Rec104, Rec114, Mei4, and Mer2), and four that are expressed during both meiosis and vegetative growth (Rad50, Mre11, Xrs2, and Ski8) [2]. These proteins interact with each other and/or with Spo11 to form a complex referred to as the Spo11- or DSB-complex, or DSB-machinery, and participate in the Spo11 transesterase reaction that leads to the formation of a DSB (reviewed in [2]).

Meiotic DSBs are essential for meiosis; nevertheless, each break represents a potentially lethal or mutagenic DNA lesion that must be repaired before the first meiotic division (MI). As such, Spo11 catalysis is tightly regulated at the temporal, spatial, and quantitative levels. For instance, the catalysis does not normally take place until the locus has undergone replication [3], [4]. When it occurs, DSB-catalysis takes place preferentially at loci referred to as DSB hotspots rather than randomly throughout the genome [5]–[7]. The number of breaks catalyzed per meiosis is also developmentally programmed; in yeast or mammals, the number is approximately 150–250 per meiosis, whereas in *Drosophila*, it is about 25 [6]–[10].

Maintaining the number of meiotic DSBs at the developmentally programmed level would require both positive and negative means of regulating break formation. Although much is known about the genetic requirements for DSB formation [2], factors and mechanisms involved in monitoring the extent of breakage and/or limiting the number of breaks remain largely elusive. Recent studies suggested a role for the mammalian ATM kinase and its *Drosophila* and budding yeast homologs, *tefu+* and *TEL1*, respectively, in down-regulating meiotic DSB formation [8], [9], [11]. These proteins are members of the ATM/ATR family of conserved signal transduction kinases involved in fundamental DNA/chromosomal processes such as DNA replication, DNA damage repair, recombination, and checkpoint regulation [12], [13]. They also play a key role(s) in many essential meiotic processes including interhomolog bias in DSB repair [14], meiotic recombination checkpoint regulation [15], and sex chromosome inactivation in mammals [16].

Here we present evidence that Rec114, an evolutionarily conserved Spo11-accessory protein and an essential component of the meiotic DSB-machinery [2], is a direct target of Tel1/Mec1, the budding yeast ATM/ATR homologues. Several Spo11-accessory proteins are proposed to be anchored at the chromosome axes and interact transiently with DSB hotspots at chromatin loops to promote cleavage [17]–[21]. Tel1/Mec1 phosphorylation of Rec114 upon DSB formation down-regulates its interaction with DSB hotspots and leads to reduced levels of Spo11 catalysis. Further analyses showed two additional means of down-regulating Rec114: synapsis associated removal at the onset of pachytene, as previously observed [17], [22], and Ndt80-dependent turnover. We propose a model whereby multiple means of regulating Rec114 activity contribute to meiotic DSB homeostasis in maintaining the number of breaks at the developmentally programmed level.

## Results

### Rec114 is a Tel1/Mec1 target

Budding yeast Tel1 and Mec1, like their mammalian counterparts, ATM and ATR, are serine/threonine kinases [23]. These kinases preferentially phosphorylate their substrates on serine (S) or threonine (T) residues that precede glutamine (Q) residues, so called SQ/TQ or [S/T]Q motifs. Many known targets of the ATM/ATR family proteins contain [*S*/T]Q *c*luster *d*omains (SCDs), defined as a region where three or more SQ or TQ motifs are found within a tract of 100 residues or less [24].

**Figure 1:**
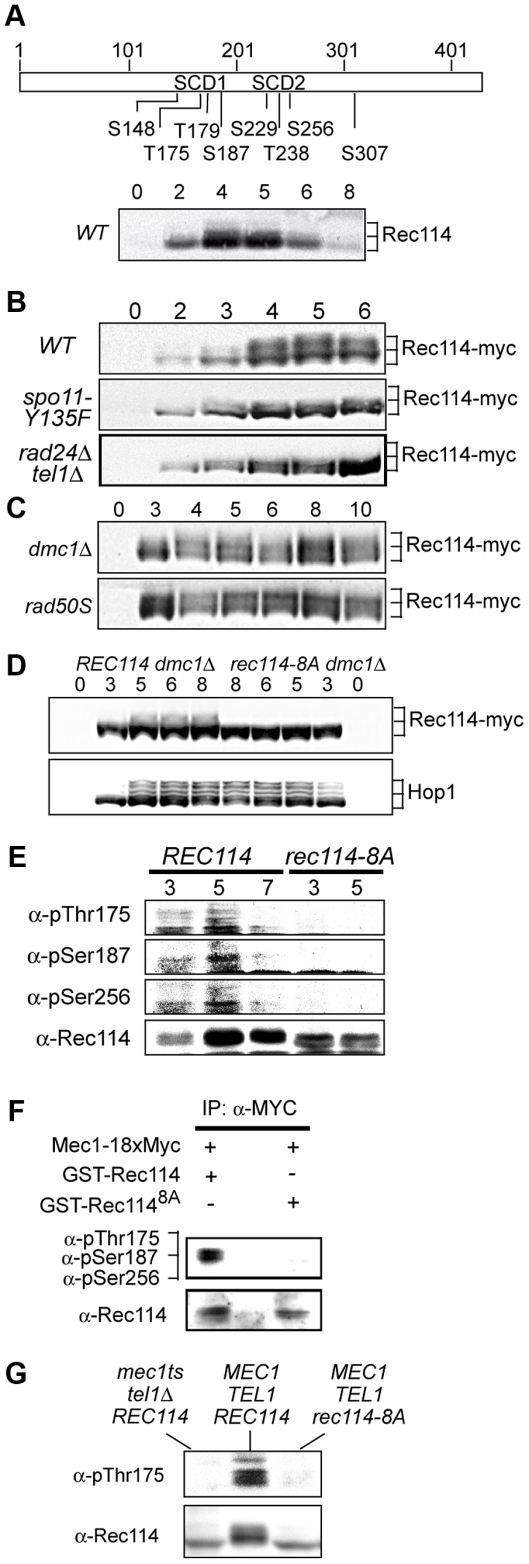
Rec114 is a DSB dependent Tel1/Mec1 target. **A**. Schematic representation of Rec114 with the locations of eight [S/T]Q motifs. S: serine, T: threonine, SCD: [S/T]Q Cluster Domain. Below: Slower migrating Rec114 species revealed in Western blot analysis using polyclonal α-Rec114 antibodies. **B–D**. Samples from indicated genotypes were collected at the specified time points and subjected to a Western blot analysis using α-Myc or α-Hop1 antibodies. **E**. Samples from *REC114* and *rec114-8A* cultures were collected at 3, 5, and 7 hours after induction of meiosis, and subjected to immunoprecipitation using α-Rec114 antibodies. The resulting precipitates were separated in SDS gels and immunoblotted using three phosphos-specific antibodies (α-pThr175, α-pSer187, α-pSer265), or α-Rec114 antibodies. **F**. *In vitro* kinase assay using immunoprecipitated Mec1-myc18 and purified GST-Rec114 and GST-Rec114^8A^ in the presence of “cold” ATP. Samples were separated in SDS gels and immunoblotted using a cocktail of α-pThr175, α-pSer187, and α-Ser265 antibodies or α-Rec114 antibodies. **G**. Samples from indicated genotypes were collected 5 hours after induction of synchronous meiosis and subjected to Western blot analysis using α-pThr175 or α-Rec114 antibodies.

As a means to investigate a role of Tel1/Mec1 in regulating DSB formation, we explored the possibility that they might directly phosphorylate one or more of the nine Spo11-accesssory proteins mentioned above. Rec114, an evolutionarily conserved meiosis specific chromosomal protein, was the most likely target with eight SQ/TQ consensus phosphorylation sites, seven of which are found in two clusters, referred to as SCD1 and SCD2 (Figure 1A). Western blot analysis using polyclonal α-Rec114 antibodies [17] revealed the appearance of slower migrating Rec114 species (Figure 1A). The putative phosphorylated isoform(s) of Rec114 was more prominent in a strain expressing a tagged version of *REC114, REC114-13xMYC* (Figure 1B “WT”). The tagged version also persisted for longer, showing that despite conferring full spore viability the tag changed some of Rec114's characteristics (see below). In both *REC114* and *REC114-13xMYC* strains, the slower migrating species became prominent by 4 hours, corresponding to meiotic prophase in the current experimental condition [14].

DSBs formed by Spo11 activates Tel1/Mec1, which in turn, directly phosphorylate a number of targets including H2AX, Sae2/Com1, (the ortholog of human CtIP), Hop1, and Zip1 [14], [25]–[27]. To test whether the Rec114 phosphorylation was also dependent on meiotic DSBs, we assessed the effect of *spo11-Y135F*, a catalytically inactive allele of *SPO11* [1]. The gel shift was not detected in protein from *spo11-Y135F* strains, indicting it is dependent on DSB formation (Figure 1B).

Next, we tested the dependence of the Rec114 mobility shift on *TEL1/MEC1*. To this end, we assessed Rec114 migration patterns in a *rad24Δ tel1Δ* strain. In a *rad24Δ tel1Δ* strain, the Tel1/Mec1 signaling is down-regulated to a level comparable to that in *mec1Δ tel1Δ* cells kept viable by a suppressor mutation, *sml1Δ*; however, *rad24Δ tel1Δ* cells do not exhibit the severe meiotic progression defect observed in the latter [14]. We found that Rec114 mobility shift was reduced in a *rad24Δ tel1Δ* background (Figure 1B). The reduction was also observed at the restrictive temperature in a *tel1*Δ strain carrying the temperature sensitive *mec1-4* allele [28] (Figure 1G).

Defects in meiotic recombination or synapsis activate Tel1- or Mec1- checkpoint response [12], [14], [15], [26], [27], [29]. In *rad50S, mre11S* (“*S*” for separation of function), or *com1Δ/sae2Δ* backgrounds, Spo11 remains covalently bound to the break ends, preventing their further processing. Accumulation of unprocessed meiotic DSBs in these mutants triggers a *TEL1*-dependent checkpoint response [30]–[32]. Elimination of the meiotic recombinase Dmc1, on the other hand, leads to accumulation of hyper-resected break ends that are loaded with single strand DNA (ssDNA) binding proteins and activates a *MEC1*-mediated checkpoint response [15], [33]. During Tel1- or Mec1-checkpoint response, a number of targets, including Hop1 and Com1/Sae2, remain hyper-phosphorylated, reflecting the increased kinase activity of Tel1/Mec1. We found that both the extent and duration of Rec114 mobility shift seemed also enhanced in a *rad50S* or *dmc1Δ* background (Figure 1C), consistent with the possibility that Rec114 might be a target of Tel1/Mec1.

To further address the role(s) of Tel1/Mec1 in Rec114 mobility shift, we examined its migration pattern in a strain expressing a *rec114* allele, *rec114-8A*, where all of the S or T residues of the eight Tel1/Mec1 consensus sites were replaced by a non-phosphorylatable alanine (A). We found that Rec114 mobility shift was abolished in a *rec114-8A dmc1Δ* strain (Figure 1D), indicating that the observed shift is due to a modification(s) at one or more of the eight Tel1/Mec1 consensus sites.

To confirm *in vivo* phosphorylation of Rec114 at a specific residue(s) during normal meiosis, we generated phospho-specific antibodies against three of the eight ATM/ATR consensus sites in Rec114. T175 and S187 were chosen based on their biological relevance (Table 1; see analysis below); S265 was selected using a software tool that predicts kinase-specific phosphorylation sites (GPS 2.1; Supporting Online Material). Using these phospho-specific antibodies, we performed Western blot analyses on samples taken from strains expressing either WT or the non-phosphorylatable allele, *rec114-8A*. The results showed that each of the three phospho-specific antibodies generated signals in the WT samples but not the *rec114-8A*, confirming *in vivo* phosphorylation of Rec114 at these three sites (Figure 1E). Finally, we demonstrated that purified Mec1 could directly phosphorylate one or more of the three confirmed *in vivo* Rec114 phosphorylation sites *in vitro* (Figure 1F). Taken together, we conclude that Rec114 is a DSB dependent target of Tel1/Mec1 during normal meiosis.

**Table 1:** Spore viability of the different *rec114* alleles in various genetic backgrounds.

Relevant Genotype^2^	None^3^	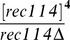	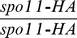	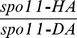	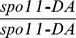	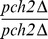
***REC114*** ** Allele** 1						
*REC114*	0.98*	0.99*	0.98	0.78	0.30	0.99
*8A*	0.99*	0.99*	0.98	0.80	0.28	0.97
*8D*	0.92*	0.68*	0.29	0.003*	<0.01	0.28*
*T175D, S187D*	0.95*	0.72	0.55	ND	ND	ND
*T175D, T179D, S187D*	0.93*	0.69	0.48	ND	ND	ND
*T175E, T175E, S187E*	0.95*	0.69	0.51	ND	ND	ND

Spore viability was assessed following 2 day incubation on sporulation medium (SPM) plate at 30°C. Generally, 160 spores were scored for each strain except for those with (*) where 320 spores were analyzed. Viability was indicated as the fraction of viable spores over the total dissected. Abbreviations: T; threonine, S; serine, A; alanine, D; aspartic acid, E; glutamic acid, ND; not determined.

1 Nature of mutations in *rec114* alleles analyzed.

2 Relevant genotypes of the strains to which *REC114*, *rec114-8A*, or the four different *rec114*-phosphomimetic alleles in the “*REC114* allele” column were introduced to assess potential genetic interaction(s).

3 Homozygous diploids expressing the indicated *REC114* or *rec114* alleles in an otherwise WT background.

4 Heterozygous diploids expressing a single copy of the indicated *REC114* or *rec114* alleles; the other allele is *rec114Δ*.

### Synthetic interaction between *rec114*-phosphomimetic and *spo11*-hypomorphic alleles

To investigate function(s) of Tel1/Mec1 phosphorylation of Rec114, the effect of mutating the S or T residues of the eight Tel1/Mec1 consensus sites was examined. We began the analysis with two *rec114* alleles, *rec114-8A* or *rec114-8D*, where the eight S or T were mutated to either a non-phosphorylatable alanine (A) or to a phospho-mimetic aspartic acid (D) residue, respectively. Spore viability of *rec114-8A* diploids was comparable to that of *REC114* in all genetic backgrounds tested (Table 1). *rec114-8D*, in contrast, conferred haploinsufficiency and synthetic interactions with mutations that confer either a reduction in DSB-catalysis (e.g. *spo11-HA* and *spo11-DA*) [34] or sensitivity to such reduction (e.g. *pch2Δ*) [35] (Table 1). Thus, constitutively mimicking Tel1/Mec1 phosphorylation might be deleterious to meiosis. Alternatively, the effect might be due to protein misfolding caused by the introduction of eight closely spaced negative charges, which might have led to its degradation. Although we cannot rigorously rule out the latter, it appears unlikely, given that chromatin bound Rec114^8D^ is more abundant than Rec114 (see analysis below), and also because replacing as few as two (T175 and S187) of the eight consensus sites with a phosphomimetic residue confers a *rec114-8D* like phenotype with respect to haploinsufficiency and synthetic interaction with *spo11*-hypomorphic alleles (Table 1). Notably, T175 and S187 of Rec114 are confirmed *in vivo* phosphorylation sites (Figure 1E).

### Rec114 phosphorylation down-regulates Spo11 catalysis

**Figure 2:**
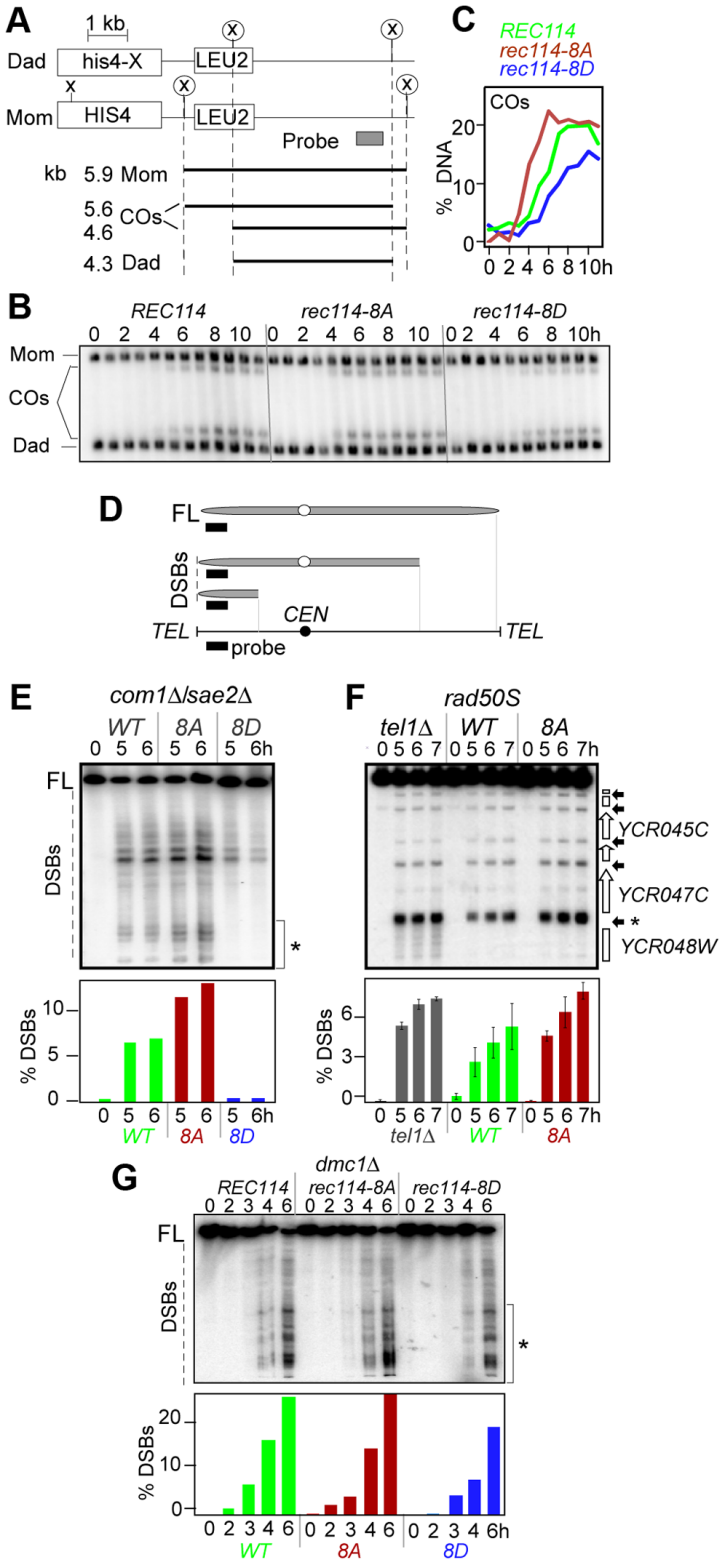
Effect of *rec114-8A* and *rec114-8D* on levels of COs and DSBs. **A**. Physical map of *HIS4-LEU2* locus showing relevant XhoI restriction sites (X) and the probe used for Southern analysis [36]. Parental homologs “Mom” and “Dad” and the two CO-products are distinguished via restriction polymorphism (circled X). Sizes and identities of species analyzed in (B) are as indicated. “COs”: interhomolog crossover products. **B**. Southern blot analysis of COs in *REC114*, *rec114-8A*, and *rec114-8D* strains. The analysis was performed as described in panel **A** and Materials and Methods. **C**. Quantification of COs in the gel shown in panel **B**. **D**. Mapping of meiotic DSBs in ChrIII by PFGE followed by indirect labeling of one chromosome end using *YCL064C*/*CHA1*. FL: full-length intact chromosomes. DSBs: linear chromosome fragments extending from the labeled end to the site of a break. **E**. PFGE of whole chromosomes probed with the *YCL064C/CHA1* probe from *REC114, rec114-8D*, and *rec114-8A* strains in a *com1Δ/sae2Δ* background; the region of the gel used for DSB quantification is indicated by brackets on the right of the gel. Quantitative analysis of the PFGE/Southern gel is presented below. **F**. Southern blot analysis of the region around the *YCR047C YCR048W* DSB-hotspot. Samples were digested with *AseI* restrictive enzyme and probed with *YCR048W* to assess DSB levels in a *REC114*, *rec114-8A*, or *tel1Δ* strain in a *rad50S* background. Quantitative analysis was performed based on the signal associated with the DSB-hotspot located within the *YCR047C* promoter (*). **G**. PFGE of whole chromosomes probed with the *YHL039W* probe from *REC114, rec114-8A*, or *rec114-8D* strains in a *dmc1Δ* background; the region of the gel used for DSB quantification is indicated by brackets on the right side of the gel.

The synthetic spore lethality interaction between *rec114*-phosphomimetic and *spo11*-hypomorphic alleles, which are known to confer sublethal reductions in crossover (CO) levels [34] (Table 1), suggested that the combined effects of the mutations may result in a lethal deficit in CO-formation. To test this, we assessed the effect of *rec114-8D* on CO-levels at the well characterized *HIS4-LEU2* artificial meiotic recombination hotspot (Figure 2A) [36]. *rec114-8D* conferred a delay in the accumulation of COs, and about 25% reduction in the final level of COs; in *rec114-8A*, the level of COs was comparable to WT but they appeared earlier (Figure 2BC).

A reduction in CO-levels can result from either insufficient DSB levels and/or a defect(s) in CO homeostasis [34]. CO homeostasis refers to the notion that CO-levels tend to be maintained at the expense of noncrossovers (NCOs), and is, in part, based on the observation that strains expressing *spo11*-hypomorphic alleles exhibited only a modest reduction in the levels of COs despite the fact that their DSB levels, assessed in a *rad50S* background, were significantly lower than WT [34]. To determine whether the reduction in CO-levels in a *rec114-8D* strain was due to a defect in break formation and/or CO homeostasis, we measured DSB levels in a *rec114-8D com1Δsae2Δ* or *rec114-8D rad50S* strain using pulsed field gel electrophoresis (PFGE)/Southern analysis (Figure 2D; data not shown). The results showed that *rec114-8D* confers a dramatic reduction in the levels of DSBs on three different chromosomes examined, ChrIII, V, and VIII (Figure 2E; Figure S1 ABC; data not shown). We conclude that the modest reduction in CO-levels in a *rec114-8D* strain is likely due to a reduction in DSB levels, and that the observed synthetic interaction between *rec114*-phosphomimetic and *spo11*-hypomorphic alleles (Table 1) may result from additive impact of the two mutations on insufficient DSB-catalysis.

The above observations suggest that Tel1/Mec1 phosphorylation of Rec114, mimicked in *rec114-8D*, down-regulates DSB formation. If so, the absence of the phosphorylation in *rec114-8A* should lead to an increase in DSB levels, assuming that no other mechanism was acting redundantly. Indeed, a substantial increase could be observed for break sites near *YCL064C* or *YCR048W* on ChrIII (Figure 2EF). The extent of the increase was comparable to that observed in *tel1Δ*, a mutant reported to cause an increase in DSB levels [11]. Since Rec114 is a target of Tel1 and/or Mec1 (above), the latter suggests that Rec114 is likely to be a key target in mediating Tel1 negative regulation in DSB levels. Unlike *rec114-8D*, whose negative effect on break levels was obvious at all break sites analyzed on ChrIII, V, and VIII, we were only able to document the much subtler positive effect of *rec114-8A* or *tel1Δ* on ChrIII with this technology (Figure 2EF; Figure S1D–E and data not shown).

The dramatic effect of *rec114-8D* suggests that phosphorylation of some or all of the sites mutated is sufficient to strongly reduce Spo11 catalysis. The comparably modest increase in *rec114-8A* mutants, where Rec114^8A^ is insensitive to Tel1/Mec1 negative control via phosphorylation at these sites, suggests that Rec114^8A^ might mainly cause repeated cleavage by the same activated DSB machine near the break on the same chromatid, which would hardly increase the DSB signals measured by Southern; alternatively, it may point to the existence of additional mechanism(s) limiting break formation, and that it/they is/are yet to be discovered.

Unexpectedly, we found that the negative effect of *rec114-8D* on break level was notably attenuated in a *dmc1Δ* background compared to *rad50S* or *com1Δ/sae2Δ* (Figure 2G; data not shown). In a *rec114-8D dmc1Δ* strain, DSB levels reached about 75% of a *REC114 dmc1Δ*. In a *RAD50 DMC1* background, the effect of *rec114-8D* was intermediate, between *rad50S/com1Δ/sae2Δ* and *dmc1Δ* (Figure S2). These observations show that the control of DSB formation is likely multi-layered and that feedbacks in addition to that by Rec114 phosphorylation exist.

### Rec114 phosphorylation leads to a genome-wide reduction in DSB levels

**Figure 3:**
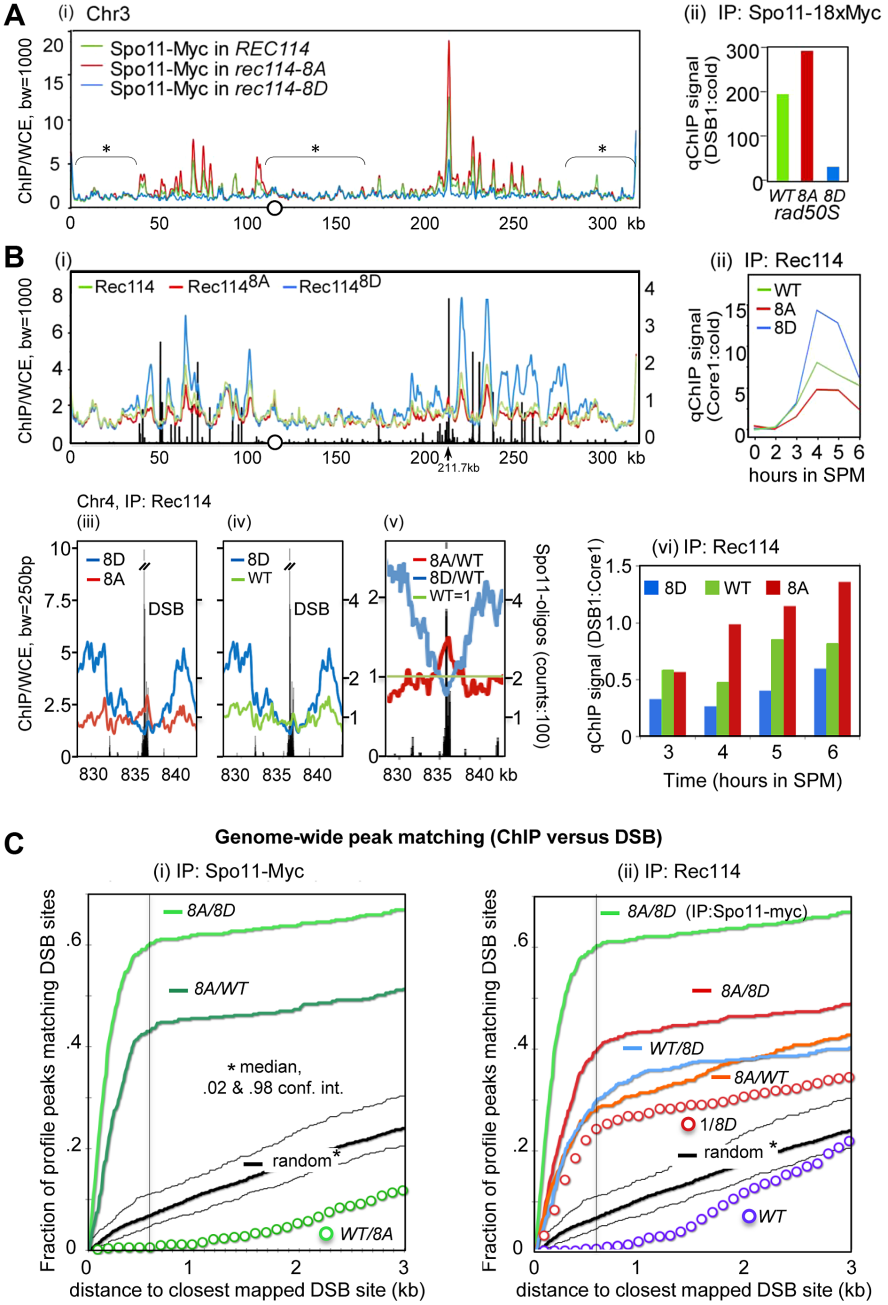
Rec114 phosphorylation down-regulates Spo11 catalysis and Rec114-DSB hotspot association. **A**. (i) Spo11-myc ChIP-chip profiles of *REC114* (green), *rec114-8A* (red), and *rec114-8D* (blue) in a *rad50S* background for ChrIII. The centromere is denoted by a circle. For all ChIP-chip profiles presented in this work, ChIP/whole-cell extract (WCE) signal intensity was plotted against the chromosomal position after smoothing (bandwidth as indicated) and after decile normalization [17]. Brackets with stars label background peaks that become aligned among the profiles by this normalization. Cells were collected 6 hours after transfer to SPM, when the DSB level in a *rad50S* strain is near its maximum. (ii) qPCR results of ChIP of Spo11-myc in *REC114, rec114-8A*, and *rec114-8D* at the *YCR047C* DSB-hotspot located at position 211.7 kb on ChrIII [17]. **B**. (i) Rec114 –ChIP-chip profiles in *REC114* (green), *rec114-8A* (red) and *rec114-8D* (blue) for ChrIII. Black bars: Hotspot positions [5]–[7]. (ii) qPCR time course of Rec114 -ChIP at a previously characterized axis site, located at 219.5 kb [17]. (iii, iv, v) Magnified views of a typical strong hotspot on ChrIV: *REC114* (green), *rec114-8A* (red), *rec114-8D* (blue), Spo11-oligo counts from [7](black bars). In (v) all profiles were normalized by wild type, as an example for the mirror-like behavior of phospho-mimicking versus non-phosphorylatable Rec114 at DSB-hotspots. (vi) qPCR time course of Rec114-ChIP, at a hotspot (211,7 kb) and an axis site (219 kb) on ChrIII, expressed as ratio of hotspot/core to demonstrate that all three strains increase Rec114 hotspot occupancy relative to its axis binding as a function of time. Notably the extent of increase is greatest in *rec114-8A*, followed by *REC114*, and then *rec114-8D*. **C**. (i, ii) Genome wide correlation between DSB-hotspots and peaks of Spo11-myc and Rec114 ChIP-chip profiles: Both plots describe how well the 500 strongest peaks of a certain profile colocalize with the 500 strongest DSBs mapped by [7] (see also Method section). The cumulative fraction of peaks of a specified profile is plotted against the distance from the nearest DSB-cluster (in kb). For example, over 60% of Spo11-myc, *rec114-8A*/*8D* peaks are within 600 bp of one of the 500 strongest DSBs (600 bp distance marked with black line for convenience). A random model would predict only 7% of overlaps under these conditions. (i) Spo11-myc ChIP-chip profile analysis in the *rad50S* background: *rec114-8A*/*rec114-8D* (8A/8D), *rec114-8A*/*REC114* (8A/WT), *REC114*/*rec114-8A* (WT/8A), random model and 2%, 98% percentiles (black). (ii) Rec114 ChIP-chip profile analysis in a *RAD50 DMC1* background: Rec114^8A^/Rec114^8D^ (8A/8D, red)), Rec114^WT^/Rec114^8D^ (WT/8D), Rec114^8A^/Rec114^WT^ (8A/WT), 1/Rec114^8D^ (1/8D) Rec114/1 (WT), random model and 2%, 98% percentiles (black). For comparison, Spo11-myc in the *rad50S* background of *rec114-8A*/*rec114-8D* (8A/8D; bright green) is included.

As an independent means of assessing the effect of Rec114 phosphorylation on DSB levels, we performed a genome-wide Spo11-chromatin immunoprecipitation (ChIP) on CHIP assay (here on referred to as ChIP-chip), which confers greater resolution and offers easier normalization than a Southern blot based analysis (e.g. [7], [37]). In constructing the required strains for the analysis, we took into account the potential genetic interaction between various epitope tags of Spo11 and *rec114* alleles as suggested by reduced spore viability of strains expressing tagged versions of either protein (Table 1; data not shown). We introduced the untagged versions of *REC114*, *rec114-8A*, or *rec114-8D* alleles into a *rad50S* strain expressing *SPO11-18xMYC*. Unlike *spo11-6xHIS-3xHA*, the *SPO11-18xMYC* did not affect spore viability of *rec114-8D* strains (data not shown). Spo11-myc ChIP was performed without the use of formaldehyde (FA) cross-linking to enrich for Spo11 proteins that had remained covalently bound to the break ends upon DNA-cleavage. To ensure the highest degree of comparability between the three *REC114/rec114* allele backgrounds, the experiments were performed strictly in parallel for all steps from culturing to the final analysis. The resulting profiles of covalently bound Spo11 in the three backgrounds reproduced the published DSB hotspot profiles [7] with great precision (Figure 3A). A small fraction of signals, typically near telomeres and within pericentric regions, however, are not DSB specific, but identical among the three profiles (Figure 3A, areas denoted by *); these were used to superimpose the profiles (decile normalization, [17], Materials and Methods). Importantly, the three aligned profiles differ in the amplitude of hundreds of sharply defined positions in an almost invariable pattern: Spo11 signal in *rec114-8A* is higher than in wild type, while Spo11 in *rec114-8D* is strongly reduced (Figure 3A; Figure S3).

The results of statistical evaluation of the differences in these peaks is presented in Figure 3C. The following prediction was tested in this analysis: If DSB formation was indeed reduced in *rec114-8D* relative to *rec114-8A*, then the ratio of the Spo11 profiles of *rec114-8A* over *rec114-8D*, (hereon referred to as 8A/8D), should define DSB sites. In fact, the correlation between DSB hotspots and the 8A/8D peaks should be greater than that of not-normalized profiles. Indeed, profiles of these ratios identify near 100% of the published DSB hotspots (eg. Figure S3 A,D). When peaks of the ratio of these profiles were compared to the mapped hotspots at a resolution of 600 bp, >97% of the 1200 strongest Spo11 8A/8D peaks matched one of the 3600 DSB sites [7], (p<10^−40^, Figure S4A). The same was true for smaller selections; 62% of 500 strongest 8A/8D sites matched one of the 500 strongest DSB sites (p<10^−40^, Figure 3C), while 76% of 100 8A/8D matched 100 DSBs (p<10^−20^, Figure S4B). More detailed results showing the cumulative curves of distances compared to a null hypothesis (random) are provided in Figures 3Ci and Figure S4A,B. Although there are some peaks in the Spo11 profiles, where 8D>8A, less than 1% of the 500 strongest 8D/8A match the 500 DSBs, a strong anti-correlation (p<10^−6^) that excludes that there is significant 8D>8A at DSB sites (data not shown). Even for the smaller difference between WT and 8A, WT/8A produces a clear anti-correlation (Figure 3Ci). Being independent of decile or any other normalization, this analysis indicates that Spo11 catalysis at nearly all known hotspots is attenuated in the phospho-mimicking *rec114-8D* background. Furthermore, the degree of attenuation is roughly proportional to the hotspot strength in that the 100 strongest DSB peaks correspond to the 100 strongest Spo11 8A/8D peaks, whereas the 500 strongest DSB peaks to the 500 strongest Spo11 8A/8D peaks.

Analysis of the smaller differences between Spo11 profiles in *rec114-8A* and in *REC114* by 500×500 comparison (500 hottest DSB hotspots against 500 strongest 8A/WT peaks) also produced a significant, although somewhat weaker, correlation (p<10^−40^, Figure 3Ci). We thus confirm with high significance, that Spo11 signals in the non-phosphorylatable *rec114-8A* are more abundant than in the wild type background, at least for the 500 strongest hotspots genome wide. The effect of *rec114* mutations on the extent of Spo11 catalysis was confirmed further by qPCR analysis at a strong DSB site (*YCR047C*, Figure 3Aii). Taken together, these results strongly suggest genome-wide down-regulation of Spo11 catalysis by phosphorylation of Rec114, at least in the *rad50S* background.

### In addition to axis-site binding, Rec114 also shows phosphorylation-sensitive interactions with DSB hotspots

Rec114 is a meiotic chromosome axis protein whose recruitment to the chromosomes is essential for Spo11 catalysis [17], [20], [22]. To test whether Tel1/Mec1 phosphorylation might down-regulate Spo11 catalysis by affecting Rec114's association with certain chromosomal positions, we performed genome wide Rec114 ChIP-chip analysis in strains expressing untagged versions of Rec114, Rec114^8A^ or Rec114^8D^ using a polyclonal antibody raised against Rec114 [17].

The analysis of Rec114 ChIP-chip after 4 hours in SPM showed enrichment of Rec114 at chromosome axes located nearby strong DSB hotspots (Figure 3Bi) as shown previously [17], [21]. Similar to the Spo11 profiles, the three Rec114 profiles became perfectly superimposed after decile normalization for many DSB-unspecific low signal peaks (Figure 3Bi). Within DSB-rich domains of ChrIII, signals at axis sites were strongest for Rec114^8D^, followed by Rec114 and then Rec114^8A^ at axis sites. This relationship was confirmed by qChIP at one axis site over a meiotic time course (Figure 3Bii). Thus, Rec114-axis association appears to correlate negatively with DSB levels. We conclude that the reduction in DSB levels in a *rec114-8D* strain is not due to defects in Rec114^8D^ -axes interaction.

RMM and other Spo11 accessory proteins are proposed to be anchored at the chromosome axes and interact transiently with DSB hotspots at chromatin loops to promote cleavage [17]–[21]. Given the apparent excess of Spo11-accessory proteins relative to the number of breaks catalyzed (e.g. [20]), such transient interaction is expected to manifest as small peaks near hotspots interspersed in a landscape of prominent axis signals. Indeed, for the hyperactive Rec114^8A^ protein, nearly all of the strong DSB hotspots show small peaks overlapping the hotspots (Figure 3Bi, at 211.7kb; Figure 3Biii, v, Figure S5). These DSB associated peaks are stronger in Rec114^8A^ than in wild type and are typically absent in Rec114^8D^. At strong hotspots, the profiles reversed their order noted above and become Rec114^8A^>Rec114>Rec114^8D^, although Rec114^8D^ strongly dominates at the immediately adjacent axis sites (Figure 3Biii, v, Figure S5). Among the 35 strongest hotspots (as defined in [7]), 33 of them presented Rec114^8A^>Rec114^8D^ (p<1.6×10^−17^), and all but one overlapped with local Rec114^8A^ maximum in the DSB cluster (e.g. Figure 3Biii, iv, v). Comparing Rec114 association with a DSB site (*YCR047C*) and its neighboring axis site as a function of time, we observed that the extent of increase at the DSB site (Figure 3Bvi) is greater than the increase at the axis site (Figure 3Bii). Furthermore, the time dependent increase in the hotspot associated Rec114 exhibited Rec114^8A^>Rec114>Rec114^8D^ (Figure 3Bvi).

Similar to arguments of the previous section, the following prediction was tested: If more Rec114^8A^ bound to DSB sites than Rec114^8D^, peaks of the ratio of the profiles Rec114^8A^/Rec114^8D^ (8A/8D) should map to DSB sites. Analysis shows that the majority of DSB-sites coincide with 8A/8D peaks (Figures S3 B, E). Indeed, comparison of the 500 strongest peaks and 500 hottest hotspots revealed a highly significant correlation (Figure 3C, p<10^−37^). Interestingly, 8A/WT and WT/8D peaks also exhibit significant correlations with DSB sites (p<10^−19^, 98% confidence interval of a random model plotted) suggesting the relation: 8A>WT>8D at DSB sites. Inversion of the DSB anti-correlated 8D profile also lead to the observed positive correlation of WT/8D (Figure 3Cii, ‘1/8D’ red circles), albeit with a weaker correlation than the 8A/8D (p<10^−7^) and WT/8D ratios (p<.04), lending solid statistical support to the interpretation Rec114^8A^>Rec114>Rec114^8D^ at the 500 strongest DSB hotspots. Selecting just 100 strongest sites produced similar significances, while selecting more hotspots (3600) results in loss of significance, as the effect of 8A becomes insignificant compared to the effect of 1/8D for weak hotspots (Figure S4).

The parallel analysis of mutations with opposite effects on DSB hotspot binding provided an opportunity to unequivocally demonstrate genome-wide associations of Rec114 with DSB sites. In addition, these mutants reveal that interaction between Rec114 and DSB sites are negatively regulated by Tel1/Mec1 phosphorylation of Rec114.

### Rec114 phosphorylation delays the onset of its *NDT80*-dependent turnover

**Figure 4:**
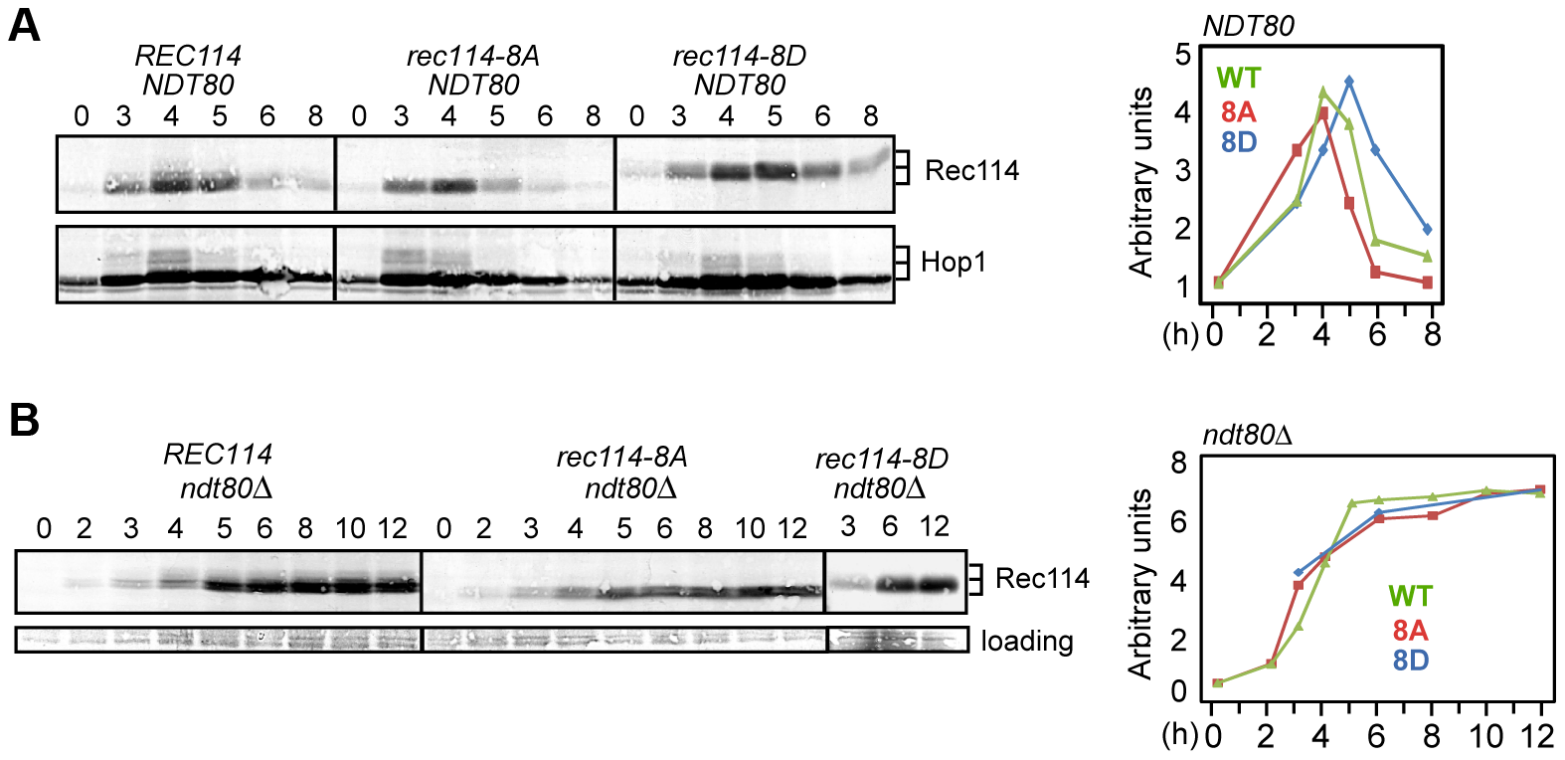
Rec114 phosphorylation delays its *NDT80*-dependent turnover. **A** and **B**. Samples from indicated genotypes were collected at the specified time points and subjected to Western blot analysis using α-Rec114 or α-Hop1 antibodies. The graphs show the level of Rec114 in the Western blot, normalized to the total Hop1 signal (A) or to the loading control (B), and expressed relative to the t = 0 sample, set to 1. In *ndt80Δ* (B), the quantification of Rec114^8D^ protein only shows timepoints 3, 6, and 12 hours.

The effects of Rec114 phosphorylation on its steady state protein levels were assessed by Western blot analysis (Figure 4) using the α-Rec114 antibody [17]. In a *rec114-8A* culture, a reduction in the protein levels, most notable at 5 and 6 hours, was observed (Figure 4A). In *rec114-8D*, protein persists longer, until the 6 and 8 hour time points. Thus, phosphorylation of Rec114 appears to increase not only its axis-association but also its steady state levels.

Ndt80 is a meiosis specific transcription factor required for pachytene exit and resolution of joint molecules (JMs). Some meiotic DSBs persist in an *ndt80Δ* background, suggesting its involvement in curtailment of break formation [38], [39], or a failure to repair some DSBs. To determine whether Ndt80 affected the stability of Rec114, we repeated the same Western blot analysis in an *ndt80Δ* background. Results revealed that Rec114 becomes stabilized in a *REC114 ndt80Δ* strain for at least 12 hours after transfer to SPM (Figure 4B), while it rapidly declined in *NDT80* after 5 hours (Figure 4A). Thus, timely Rec114 degradation requires Ndt80. *ndt80Δ* also prevented the degradation of Rec114^8A^ and Rec114^8D^ (Figure 4B), suggesting that the observed differences in steady state protein levels in the mutants (Figure 4A) might be caused by differential timing of Ndt80 activation.

### Rec114 phosphorylation delays its synapsis-associated removal from chromosomes

All Spo11-accessory proteins examined to date, including Rec114, are recruited to the chromosomes before the initiation of meiotic recombination, and remain chromosome-associated until Zip1 dependent homolog synapsis takes place [17], [20], [22], [40]. Zip1 is an evolutionarily conserved component of the central region of the synaptonemal complex (SC), and is required for homolog synapsis and meiotic recombination [41]–[43].

In early meiotic prophase, there is little overlap between Rec114 and Zip1; at later stages, Rec114 foci become less abundant and dimmer in synapsed chromosome regions but remain bright in unsynapsed regions of the same nucleus [17], [22]. These observations suggest that synapsis might promote the removal of Rec114 and its associated proteins Mei4 and Mer2. Combining this with the current observation that the extent of Rec114-axis association is affected by its phosphorylation status (Figure 3B) raised the possibility that Rec114 phosphorylation might affect the timing of synapsis. To address this, we performed co-immunostaining analyses of Rec114 and Zip1 using polyclonal antibodies raised against each protein (Supplementary Online Information). The experiment was conducted in an *ndt80Δ* background to exclude any influence by the *NDT80* dependent Rec114 degradation (above).

**Figure 5:**
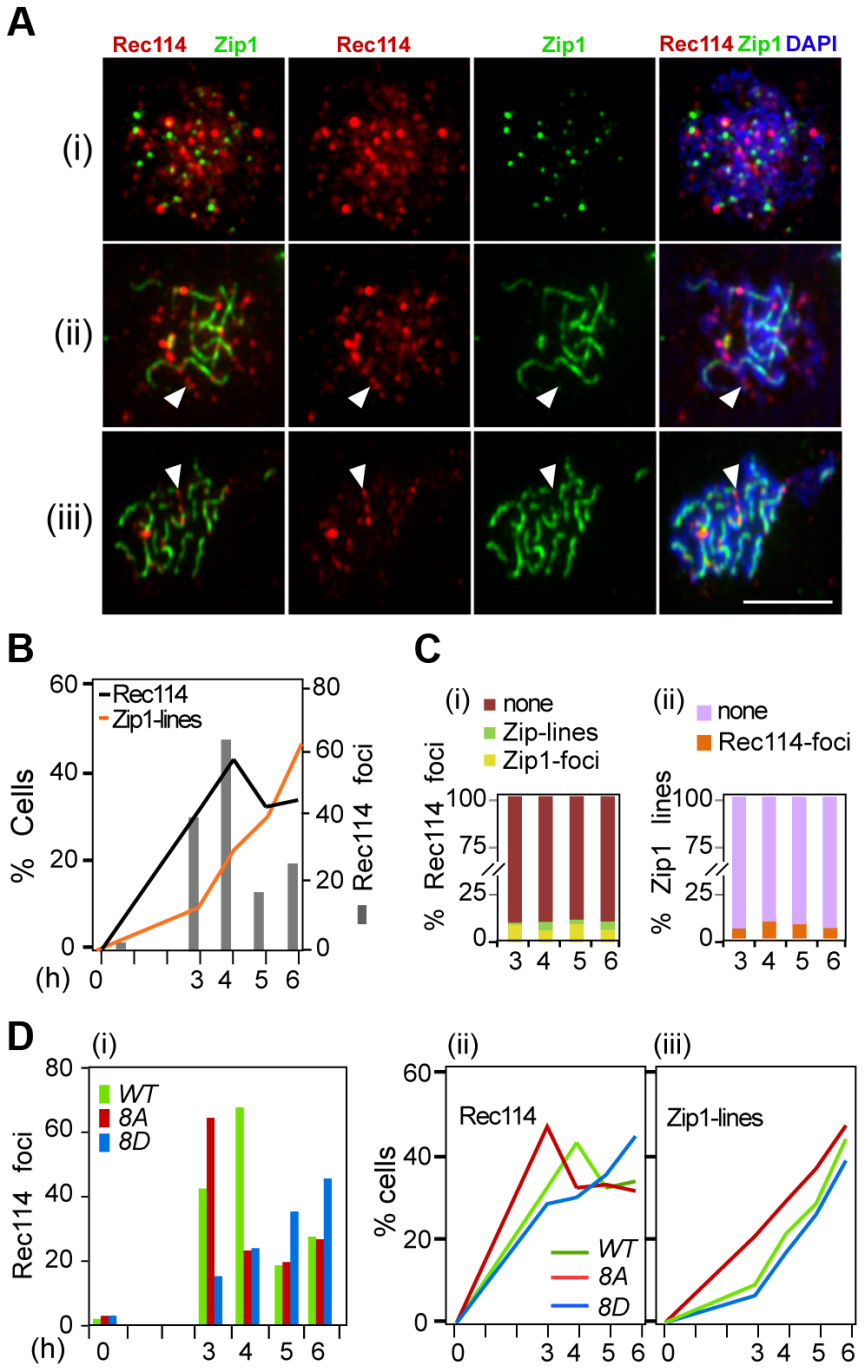
Effect of Rec114 phosphorylation on its synapsis dependent removal. **A**. Temporal and spatial dynamics of Rec114 and Zip1 localization are assessed cytologically using antibodies against each protein. Presented are representative images of cells in leptotene/zygotene (i); zygotene/pachytene (ii); and pachytene (iii). The classification was based on the extent of Zip1-polymerization. White arrowheads: examples of the mutual exclusiveness of Rec114 and Zip1 signals. Scale bar: 5 µm. **B**. The fraction of *REC114 ndt80Δ* cells with Rec114 foci (black lines) or Zip1-linear stretches (orange lines). Grey columns; the average number of Rec114 foci per cell. **C**. (i) Fraction of Rec114-foci co-localizing with either Zip1-foci (yellow) or Zip1-lines (green). For each time point, ∼500 Rec114-foci collected from ∼ *REC114 ndt80Δ* nuclei were analyzed. (ii) Fraction of Zip1-lines co-localizing with Rec114-foci in the same ∼50 *REC114 ndt80Δ* nuclei per time point analyzed in panel (i). **D**. The average number of Rec114 foci (i), fraction of cells containing Rec114 foci (ii), and fraction of cells containing Zip1-linear stretches (iii) in *REC114 ndt80Δ* (green), *rec114-8A ndt80Δ* (red) or *rec114-8D ndt80Δ* (blue) cells.

Rec114 in the *ndt80Δ* background behaved as reported [17], [22], with Rec114 foci peaking at mid prophase just before the onset of synapsis, with little or no overlap between Rec114 and Zip1 staining (Figure 5A,C). The fraction of nuclei containing Rec114^8A^ -foci decline more rapidly than Rec114, while that of Rec114^8D^ containing nuclei remain abundant until at least 6 hours in SPM (Figure 5Di, ii), consistent with synapsis being affected by the status of Rec114 phosphorylation (Figure 5D iii). These observations show that synapsis-associated dissociation of Rec114 is Ndt80 independent. Depending on the Rec114 allele and the associated DSB frequency, synapsis occurs earlier or later, entailing earlier or later Ndt80 independent Rec114 removal. In ∼30% of *ndt80Δ* cells, some strong Rec114 foci persisted up to 6 hours into meiosis (Figure S6), consistent with the stabilization of the protein in *ndt80Δ* (Figure 4). Most of the Rec114 positive *ndt80Δ* cells exhibited dimming and/or disappearance of signal along SCs and a persistent polycomplex (PC) (Figure S6i, ii), in agreement with ‘SC-decay’ in *ndt80* mutants [38]. All prominent Rec114 foci were on Zip1-free areas or in PC (Figure S6i, iii), suggesting that they might be aggregates of stripped Rec114 that cannot be degraded in an *ndt80* background. The abundance of residual Rec114 present at these late time points is consistent with the aberrantly late DSBs observed in *ndt80Δ* [39].

## Discussion

Rec114 is an evolutionarily conserved essential component of the meiotic DSB-machinery [17], [20], [22], [44]–[48]. Here we present evidence that phosphorylation of Rec114 reduces both its interaction with DSB-hotspots and DSB formation. Furthermore, it prolongs Rec114-axis association and delays the onset of *NDT80* dependent turnover, suggesting the existence of a feedback system that couples the steady state DSB levels to post-leptotene regulation of Rec114 activity.

### DSB-dependent Tel1/Mec1 phosphorylation of Rec114

Three independent studies have implicated a role of the ATM kinases in down-regulating Spo11 catalysis [8], [9], [11]. The evidence presented here implicates Rec114 as a physiologically relevant Mec1 and/or Tel1 target in this regulation. Results show a robust reduction of function for phospho-mimicking Rec114^8D^ and a subtler increase in function in the *rad50S* background for Rec114^8A^. Why are these effects not symmetric? One trivial explanation could be that introducing eight aspartic acid residues into Rec114^8D^ may render the protein partially non-functional, aside from mimicking phosphorylation. However, Rec114^8D^ is a stable protein that binds well to chromatin, excluding general protein stability-, nuclear import- or chromatin-binding defect for Rec114^8D^. Furthermore, since Rec114^8D^ behaves similar to Rec114^2D^ (T175D, S187D) in terms of reduction of DSBs, inferred based on reduced spore viability in a *spo11-HA* background (Table 1), and two aspartic acid exchanges are less likely to strongly damage the protein, we favor an interpretation involving constitutive phospho-mimicking as explanation. If so, phosphorylation is sufficient to tune down DSB formation (e.g. Rec114^8D^ or Rec114^2D^), while other effects might prevent the observation of a strong increase in break levels under constitutive “on” conditions (e.g. Rec114^8A^).

Several models (e.g. [14], [17], [49]) propose that a first negative feedback may be locally restricted to the activated DSB-machine and its surrounding chromatin loops. Phosphorylation of Rec114 would be ideally suited to mediate such a control. However, repeated cleavage of the already broken chromatid is not expected to lead to an increase of the DSB signal. Cleavage of hotspots on the intact sister chromatid could be responsible for the 20–30% increase observed by the ChIP-chip analysis in the *rad50S* background. Increased DSB formation in Rec114^8A^, even if only moderate, identifies Rec114 as a rate limiting target of negative feedback at least in the *com1Δ/sae2Δ* or *rad50s* background. On the other hand DSB formation is strongly impeded in Rec114^8D^ (or Rec114^2D^), suggesting that phosphorylation affects a critical function of Rec114. Importantly, phosphomimicking Rec114^8D^ shows a reduced interaction with DSB-hotspots suggesting a plausible mechanism explaining its reduced activity.

### Synapsis dependent removal of Rec114

Budding yeast Rec114 physically interacts with Mei4 and Mer2, two other components of the DSB-machinery, to form a complex referred to as RMM (Rec114-Mei4-Mer2) [20], [22]. RMM foci become abundant in early meiotic prophase and the proteins accumulate on DNA sequences, which organize the chromosome axis upon condensation; when chromosomes synapse, RMM foci become dimmer and eventually disappear [17], [22], an observation confirmed here in a strain expressing untagged Rec114 (Figure 5). The strong correlation between the appearance of Zip1-lines and the disappearance of Rec114 foci from the synapsed regions of the chromosomes suggests that the process of synapsis itself could be removing RMM foci. Synapsis dependent removal of RMM occurs independently of Tel1/Mec1 phosphorylation and Ndt80 (and thus protein degradation), consistent with being an independent mechanism of down regulating DSB levels.

### *NDT80* dependent Rec114-turnover

During normal meiosis, steady state levels of meiotic DSBs decrease to the background level as cells proceed beyond pachytene with complete cessation of DSBs depending on Ndt80 [39]. The Ndt80 dependent Rec114 degradation reported here (Figure 4) presents a plausible mechanistic explanation for the latter.

**Figure 6:**
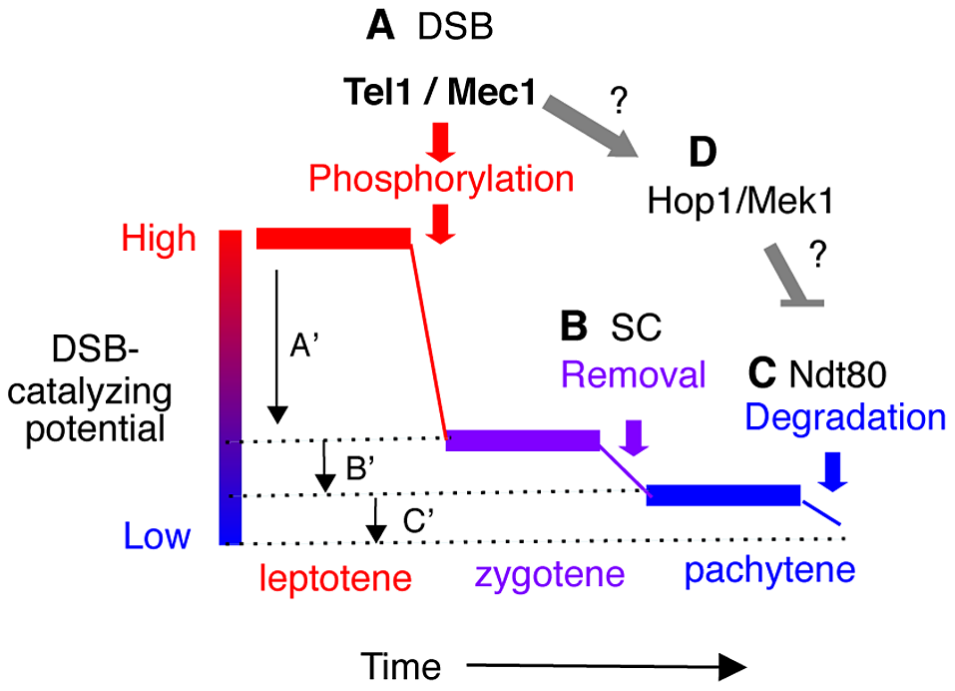
Model: Multiple mechanisms of regulating Rec114 contribute to meiotic DSB homeostasis. **A**. Tel1/Mec1 phosphorylation of Rec114 following a successful Spo11-cleavage leads to local inhibition of DSB formation near the break. Given that most of Spo11-breaks are generated during leptotene, a feedback mechanism based on successful Spo11 catalysis would be most effective during this period, contributing to a large reduction in the DSB-catalyzing potential of the cell as depicted by A′. **B**. Synapsis-dependent Rec114-removal from chromosomes during the zygotene to pachytene transition contributes to a modest reduction in the DSB-catalyzing potential of the cell as depicted by B′. **C**. Ndt80-dependent Rec114-turnover would lead to irreversible inactivation of DSB-catalyzing potential at the genome-wide level (C′). The continued DSB formation observed in *ndt80Δ* strains [39] could be attributable to the persistent low level DSB catalyzing potential. **D**. Tel1/Mec1 activation of Hop1/Mek1 checkpoint function inhibits Ndt80, which in turn, ensures that cells do not progress through meiosis I until DSB repair is complete. Involvement of Ndt80 in Rec114 degradation (Figure 4) suggests that Tel1/Mec1, depending on circumstances, might also positive regulate DSB levels by preventing irreversible inactivation of DSB machinery.

Normally, Ndt80 activation is coupled to meiotic DSB-repair and synapsis. In response to defects in either process (e.g. in a *rad50S*, *dmc1Δ* or *zip1Δ* background), Tel1/Mec1 prevent Ndt80 activation by hyper-phosphorylating Hop1/Mek1 [50]. Hop1 is an evolutionarily conserved meiotic chromosome axis protein that functions as a meiotic paralog of Rad9 in the Tel1/Mec1 signaling cascade [14]. Hyper-phosphorylation of Hop1, in turn, activates the checkpoint function of Mek1, a meiotic chromosome axis associated serine/threonine kinase, and a meiotic paralog of Rad53/Chk2 [14]. Importantly, the Tel1/Mec1-Hop1/Mek1-Ndt80 signaling pathway appears to also regulate normal meiotic progression [12], [38], [51]–[53], raising the possibility that the observed earlier or delayed onset of *NDT80* dependent Rec114 turnover in *rec114* phospho-mutants might be under Tel1/Mec1 regulation (Figure 6).

### Meiotic DSB homeostasis, the tendency to maintain similar DSB levels under different circumstances

The term “meiotic DSB homeostasis” was originally introduced to refer to the phenomenon, whereby the accumulated DSB frequency in a chromosomal region appeared to be maintained at a constant level [54]. Here, we expand the meaning to include that the break frequency might be regulated not only at the regional, but also at genome wide level. A sophisticated system controlling chromosome synapsis and recombination is expected to operate, at least, at two levels:

First, the DSB machinery needs to be “informed” about the success of a particular DSB catalysis. This local negative feedback should be limited to the immediate environment and should prevent repeated cleavage of the already broken chromatid near the break. One manifestation of this local down regulation would be DSB interference, or “competitive inactivation” of weaker hotspots, by a nearby strong hotspot [54]–[57]. We show evidence that phosphorylation of Rec114 is a key step in communicating DNA breakage to the DSB machinery via Mec1 and/or Tel1.

Second, nucleus wide (global) signaling of successful completion of homolog synapsis and meiotic DSB repair should precede irreversible global inactivation of the DSB machinery. We present evidence for two feedback based mechanisms of such regulation: synapsis dependent removal and Ndt80 dependent degradation of Rec114. Linking the progression of synapsis to down regulation of DSB formation conveniently ensures that enough DSBs have been formed to guarantee successful homology search. However, synapsis alone does not appear to lead to irreversible inactivation of the DSB machinery. This ultimate decision is instead linked to the exit from pachytene, when the activation of Ndt80 provides some guarantee that early prophase events have successfully completed.

Evidence here and elsewhere indicates that cells have the means to prevent excessive DSB formation via negative feedback [8], [9], [11]. But what happens when the break level is too low? A key feature of these nested feedback loops (Figure 6) is that a reduced Spo11 activity, independent of its cause, will delay the completion of synapsis and the onset of Ndt80 activation, and thus provide more time to accumulate breaks, even at a low rate. For instance, delayed synapsis and Ndt80 activation in the DSB poor *rec114-8D* mutant, would delay RMM inactivation, likely prolonging its active lifespan and raising the level of DSBs eventually produced. Indeed, Rec114^8D^ remained longer and in greater abundance at chromosome axes than Rec114 or Rec114^8A^. DSB homeostasis could also account for the apparent ‘catching up’ of break levels in a *rec114-8D dmc1Δ* strain (Figure 2G). For instance, defective recombination and synapsis in the mutant would severely compromise Zip1 dependent RMM removal, while *dmc1Δ* activation of Mec1-Hop1/Mek1 checkpoint response would prevent Ndt80 dependent Rec114 degradation, thus allowing Rec114^8D^ to remain active. The fact that break levels in *rec114-8D rad50S* remain low, apparently unable to catch up, suggests that DSB repair beyond *rad50S* arrest point (e.g. endonucleolytic removal of Spo11 followed by break resection) might be required to activate the synapsis and/or Ndt80 based feedback loops (Figure 6).

DSB homeostasis may contribute significantly to the relatively mild effect on spore viability of mutants (e.g. *spo11*-hypomorphs) with a low rate of DSB formation that was up to now solely attributed to CO homeostasis. But clearly, postponing the inactivation of the DSB machinery in response to problems in synapsis and break repair helps to provide more DSBs, on which CO homeostasis can act to ensure correct chromosome segregation.

## Materials and Methods

Standard yeast manipulation procedures and growth media are utilized. All strains were of the SK1 background (Table S1). Specific [S/T]Q to AQ and DQ mutations were introduced and sequenced to ensure that no additional mutations were created during the mutagenesis. Standard Western blot, Southern blot, and spread surface immunofluorescence techniques were used. Chip on CHIP and qPCR were performed as described in [17].

### Yeast strains and media

Standard yeast manipulation procedures and growth media were utilized. All strains are of the SK1 background; relevant genotypes of the strains are listed in Table S1.

### Construction of *rec114* strains

The myc13 tag from a *REC114-MYC13-HYGRO* plasmid (pNS2) was removed to generate pJC15, an integration plasmid without an epitope tag. Specific [S/T]Q to AQ or DQ mutations were introduced into either pNS2 or pJC15 utilizing the QuickChange Multi Site-Directed Mutagenesis kit (Stratagene). The entire open reading frame (ORF) of each allele was sequenced to ensure that the allele did not contain any incidental mutation(s). Each *rec114* allele was introduced into a *rec114Δ::KanMX4* haploid strain (RCY336/337), where the endogenous *REC114* gene was replaced by a kanamycin resistant gene. Transformants were identified based on their ability to grow on hygromycin plates but not on kanamycin. Southern blot and PCR analyses were performed on candidate colonies to confirm integration of a single copy of a specific *rec114-HygroMX4* allele at the endogenous locus, replacing the *rec114Δ::KanMX4* allele. Correct *rec114* haploid transformants of each allele were taken through standard yeast genetics manipulation to generate corresponding *rec114* homozygous diploid strains suitable for meiotic analyses.

### Generation of phospho-specific Rec114 antibodies

Three of the eight S/T[Q] consensus sites in Rec114, T175, S187 and S256, were selected for generation of phospho-specific antibodies. T175 and S187 were chosen based on the fact that replacing these residues with a non-phosphorylatable alanine (A) confers haploinsufficiency and synthetic interaction with *spo11* hypomorphic alleles (Table 1). S256 was chosen because it was one of the six residues within Rec114 that were predicted to be the most likely ATM/ATR phosphorylation sites (GPS2.1 software [58]). Specificity of each phospho-specific antibody was confirmed by Western blot analysis of *rec114* strains, each expressing a *rec114* allele missing a specific phosphorylation site(s).

### Synchronous meiotic time course

Induction of synchronous meiosis is carried out according to the established protocols [17], [59]. All pre-growth and meiotic time courses were carried out at 30°C except for *mec1-4^ts^ tel1Δ sml1Δ* meiosis, where the culture was kept at 23°C and shifted to 30°C 2 hours after transferring into sporulation medium (SPM).

### Protein purification and manipulation methods

*GST-REC114* and *GST-rec114-8A* plasmid-construction and protein expression were carried out as described [60]. To purify Mec1-myc18 from yeast cells, 500 ml of logarithmically growing cell cultures were subjected to 1 hour incubation with 0.1% methyl methanesulfonate (MMS) followed by Immunoprecipitation using Goat anti-myc-agarose antibodies (AbCam). Mec1-myc immunoprecipitates were mixed with reaction cocktail containing kinase buffer, cold ATP, and either GST-Rec114 or GST-Rec114^8A^. The mixtures were incubated at 30°C for 25 minutes and subjected to electrophoresis on SDS gels. Gels were transferred onto a nitrocellulose membrane and subjected to Western blot analyses using anti-Rec114 or phospho-specific antibodies.

### Western blot analysis

Whole-cell extracts (WCE) were prepared from cell suspensions in 20% trichloroacetic acid (TCA) by agitation with glass beads. Precipitated proteins were solubilized in SDS-PAGE sample buffer, and appropriate dilutions were analyzed by SDS-PAGE and Western blotting. Antibodies for Western blotting were mouse monoclonal anti-myc (1∶1000, AbCam), rabbit polyclonal anti-Rec114 (1∶1000), anti-Phospho-Rec114-S187, anti-Phospho-Rec114- T175, anti-Phospho-Rec114- S265 (1∶1000, Cambridge Research Biomedicals), goat anti-mouse IgG conjugated to horseradish peroxidase (1∶10,000; Sigma-Aldrich), and donkey anti-rat IgG conjugated to horseradish peroxidase (1∶10,000; Sigma-Aldrich).

### Southern blot analysis

Southern blot analysis following Pulse Field Gel electrophoresis (PFGE) using DNA prepared in agarose plugs or standard agarose gel electrophoresis were performed as described [61]. Exception was that the PFGE gels shown in Figure 2G and Figure S1A were run with the following modifications: initial switch time; 15 sec – final switch time; 32.5 sec, in order to better separate large chromosomes. For quantifying the level of DSBs, only the signals associated with breaks proximal to the probe was utilized to maximize the detection of chromosomes that acquired more than one break (see [3] for discussion).

### Chromatin Immunoprecipitation on CHIP (ChIPchip) and quantitative PCR (qPCR)

Rec114 and Spo11-myc chromatin immunoprecipitation (ChIP), quantitative PCR (qPCR), and microarrays hybridization/analysis were performed as described [17].

### Cytological methods

Surface spread meiotic chromosomes were prepared as described [14]. Staining was performed as described [14] with the following primary antibodies: rabbit polyclonal anti-Rec1141 (1: 100, F. Klein, MFPL), mouse monoclonal anti-HA (12CAS, 1∶100, S. Ley, NIMR), mouse monoclonal anti-MYC (9E10, 1∶100, S. Ley, NIMR goat polyclonal anti-Zip1 (1∶50, SantaCruz Biotechnology). Secondary antibodies (Invitrogen) were used at a 1∶500 dilution: chicken anti-mouse Alexa-488, anti-goat Alexa-488, chicken anti-rabbit Alexa-594. Chromosomal DNA was stained with 1 ug/ml 4,6-diamino-2-phenylimide (DAPI). Images were recorded and analyzed using a Deltavision (DV3) workstation from Applied Precision Inc. with a Photometrics CoolSnap HQ (10–20 MHz) air cooled CCD camera and controlled by Softworx image acquisition and deconvolution software.

### Significance

For comparing proportions (e.g. matching versus non-matching peaks) significance values were computed using Fisher's exact test (http://www.langsrud.com/fisher.htm).

### Statistical analysis of the Chip on CHIP

data was done as described [17]. Briefly, CEL files were converted using Affymetrix's “tiling array software” (TAS) and expressed as ChIP relative to WCE (whole cell extract) or as ChIP relative to another ChIP. The output of TAS (intensities per chromosome position) was smoothed using bandwidths between 250 and 1000 bp (ksmooth, statistic package R) and plotted for all chromosomes. For microarrays “decile normalization” was used. It is often a good choice for automatic background correction [17]. For the profiles to be compared in this work, a single correction factor was determined per profile F = 1/(0.1 percentile). These factors were usually very close to 1. After multiplying all profile intensities with their correction factors, they were precisely superimposing on all background peaks (compare Fig. 3A 0–30 kb, and 115–155 kb, see brackets with asterisk).

### Peak calling and peak matching

Peaks were called automatically as described previously [17]. To quantify the overlap between peaks of one profile and the meiotic hotspot map published by [7], we used their simplified list of hotspots, organized in 3600 blocks, listing start and end of each hotspot array and the number of mapped 5′-ends detected. We defined the distance of a peak to its nearest hotspot as the distance to the nearest edge of a hotspot block (even when the peak mapped inside the block). Most of these arrays have a very narrow width, (median 180 bp, mean 252 bp, 0.9 quantile 507 bp). In order to simulate random distributions, we generated random positions corresponding to the numbers of peaks to be tested and mapped them relative to the hotspot-blocks, the same way as the experimental data. (Simulations were repeated 100 times to obtain the 2% and 98% percentiles plotted). For example, for the comparison of the 500 highest peaks with the 500 strongest hotspots, the hotspots were sorted according to strength, the peaks were sorted according to strength and then the top 500 of each list were compared. For each of the 500 highest peaks, the distance to the nearest hotspot-block was determined and the distances accumulated and plotted.

## Supporting Information

Figure S1 Effect of *rec114-8A, rec114-8D* and *tel1Δ* on the levels of DSBs in a *com1Δ* background. A. 0, 5 and 6 hour samples from *REC114 com1Δ, rec114-8A com1Δ*, and *rec114-8D com1Δ* cultures were analyzed for the extent of chromosome breakage in ChrV and ChrVIII using *YER180C* and *YHL039W* as probes, respectively. The region of the gel used for DSB quantification is indicated by an *. B,C. Quantification of signals in the region specified in A. D. PFGE/Southern analysis of ChrIII using *YCR098C* as a probe in *tel1Δ*, *REC114, rec114-8A*, in a *com1Δ* background at the indicated time. The region of the gel used for DSB quantification is indicated by an *. E. Quantification of signals in the region specified in D.
(PDF)

Figure S2 Mimicking Rec114 phosphorylation leads to a modest reduction in DSB levels at *HIS4-LEU2* hotspots. A. (i) Representative image of a Southern analysis of *HIS2-LEU2* artificial recombination hotspot. Relevant DNA fragments are as described in Figure 2A; parental homologs “Mom” and “Dad”, the two CO-products, and DSBs. (ii) Darker exposure of the “DSB” region. B. Quantification of signals in the DSB region in A.
(PDF)

Figure S3 DSB sites match peaks of Spo11-myc and Rec114^8A^ profiles. A. Spo11-myc profile of a *rec114-8A rad50S* strain normalized (divided) by Spo11-myc profile of a *rec114-8D rad50S* strain (green, “Spo11-8A/8D”). Red bars represent Spo11-oligo counts per hotspot cluster [7] Small chromosome VI is shown as an example to illustrate genome wide colocalization between Spo11-8A/8D peaks and DSBs. B. Rec114 profile of *rec114-8A* normalized (divided) by Rec114 profile of *rec114-8D* (blue, “Rec114 8A/8D”) and *REC114* normalized by *rec114-8D* (bright green, “WT/8D”). Red bars represent Spo11-oligo counts per hotspot cluster [7]. Small chromosome VI is shown as an example to illustrate genome wide colocalization between peaks of Rec114^8A^/Rec114^8D^ and Rec114/Rec114^8D^ and DSBs. **C**. At axis sites defined by peaks of the axis protein Hop1 [17], “1” was plotted, if 8D/8A exceeded a certain threshold (0.5), while “0” was plotted otherwise. Both, groups of “1 s” and groups of 0 s” cluster together in the hot and cold DSB domains, respectively (50 axis sites). E., D., F. As in A., B., C. but on the larger chromosome IX. F. is built from 78 axis sites.
(PDF)

Figure S4 Genome wide correlation between DSB hotspots and peaks of Spo11-myc and Rec114^8A^ profiles. A. The cumulative fraction of peaks of a specified profile is plotted against the distance from the nearest DSB cluster (in kb). Results of comparison between 3600 DSB sites [7] and the 1135 strongest peaks of various profiles are presented. B. Same as in A, but the comparison was between 100 strongest DSB hotspots and 100 strongest peaks of various profiles.
(PDF)

Figure S5 Additional examples of the mirror-like behavior of Rec114^8A^ versus Rec114^8A^ at DSB hotspots. Rec114 ChIPchip profiles of *REC114* (green), *rec114-8A* (red), and *rec114-8D* (blue) are shown for selected regions in ChrVI (A), ChrIX (B), and ChrXI (C). * denotes strong DSB hotspots where Rec114 signal is highest in *rec114-8A* followed by *REC114* and then by *rec114-8D* (upper panel). Ratio between signals from each mutant over wild type (lower panel) shows that majority of DSB hotspots are loci showing 8A>WT>8D quantitative relationship, while nearby axis shows 8D>WT>8A. For very weak hotspots, however, it is difficult to discern the 8A>WT>8D relationship as the positive effect of *rec114-8A* becomes insignificant compared to the negative effect of *rec114-8D*.
(PDF)

Figure S6 Rec114 foci persist in the *ndt80* background. A. Representative images of *REC114 ndt80Δ* cells at t = 6 hours showing persistent Rec114 foci in Zip1 free regions or a PC (stars). B. Fraction of cells showing a polycomplex (PC) in *REC114 NDT80* (black columns) or *REC114 ndt80Δ* (white columns) cells as a function of time. C. Fraction of cells showing a PC at t = 6 hours in the indicated strain background. Majority of PCs contained both Zip1 and Rec114 signals (yellow).
(PDF)

Table S1 *S. cerevisiae* strains used in this study. All strains are *MATa/MATα* diploids homozygous unless specified. For *HIS4LEU2* hotspot recombination assay diploid strains JCY1193 (*REC114*), JCY1195 (*rec114-8AQ*) and JCY1197 (*rec114-8DQ*) were derived from NHY187 (*Mata, ho::hisG, leu2, ura3, HIS4-LEU2NewBamH*) and NHY285 (*Matα, ho::hisG, leu2, ura3, his4X-LEU2NewBamH-URA3*).
(PDF)

Text S1 Supplementary methods.
(DOCX)
