# Supplementary figures and images for: Budding Yeast ATM/ATR Control Meiotic Double-Strand Break (DSB) Levels by Down-Regulating Rec114, an Essential Component of the DSB-machinery
Source: PLoS Genet. 2013 Jun 27;9(6):e1003545. doi: 10.1371/journal.pgen.1003545 (PMC3694840; doi:10.1371/journal.pgen.1003545)

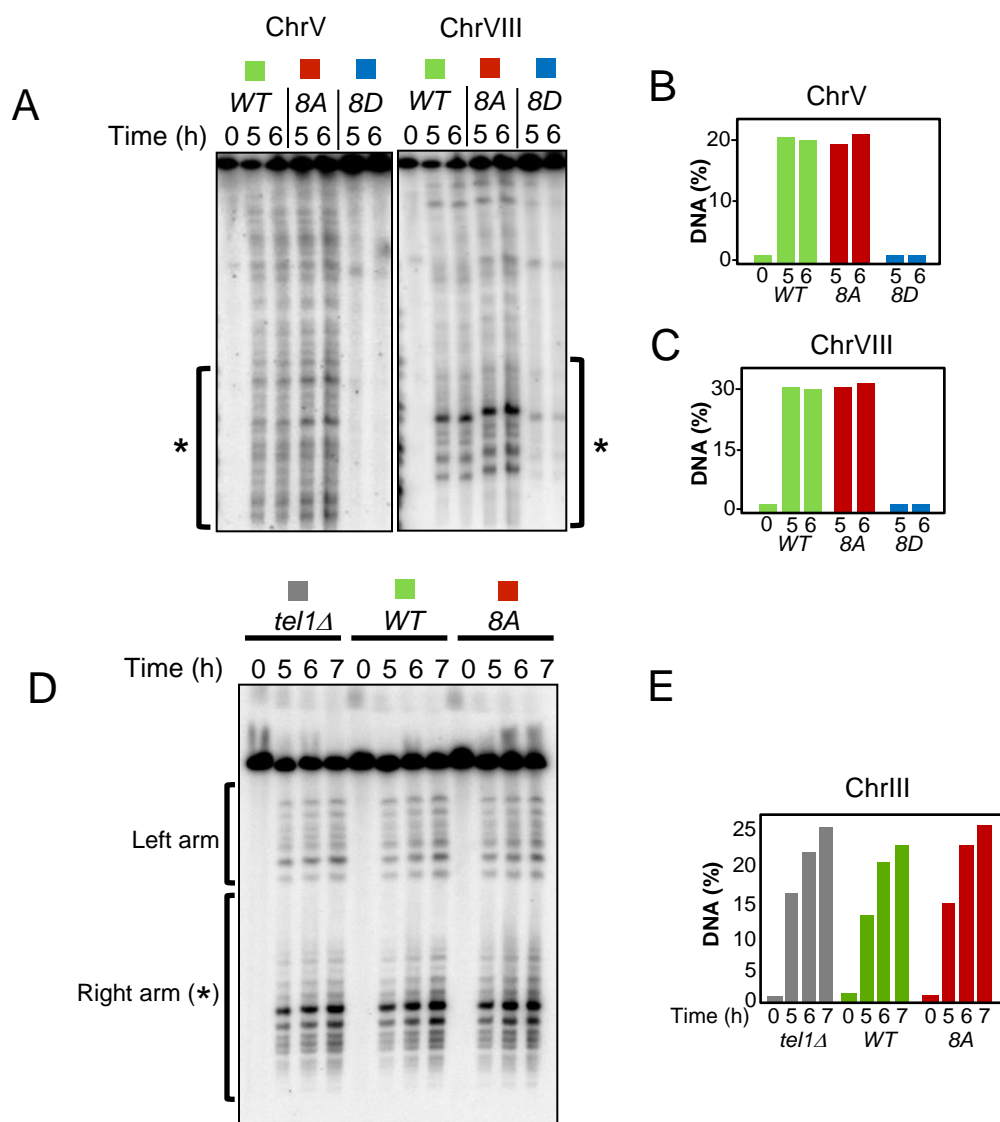

Supplement: Figure S1 — Effect of rec114-8A, rec114-8D and tel1Δ on the levels of DSBs in a com1Δ background. A. 0, 5 and 6 hour samples from REC114 com1Δ, rec114-8A com1Δ, and rec114-8D com1Δ cultures were analyzed for the extent of chromosome breakage in ChrV and ChrVIII using YER180C and YHL039W as probes, respectively. The region of the gel used for DSB quantification is indicated by an *. B,C. Quantification of signals in the region specified in A. D. PFGE/Southern analysis of ChrIII using YCR098C as a probe in tel1Δ, REC114, rec114-8A, in a com1Δ background at the indicated time. The region of the gel used for DSB quantification is indicated by an *. E. Quantification of signals in the region specified in D. (PDF) [file pgen.1003545.s001.pdf]

**A.**

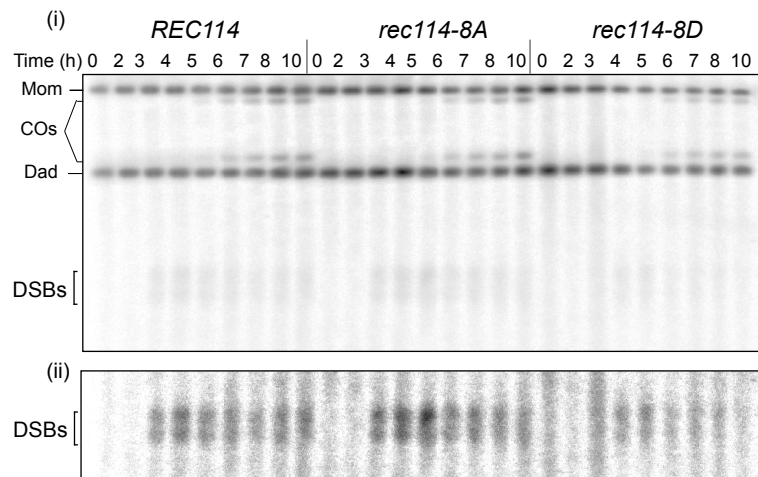

**B.**

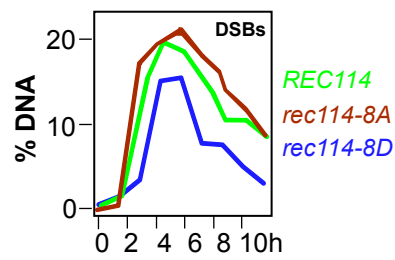

Supplement: Figure S2 — Mimicking Rec114 phosphorylation leads to a modest reduction in DSB levels at HIS4-LEU2 hotspots. A. (i) Representative image of a Southern analysis of HIS2-LEU2 artificial recombination hotspot. Relevant DNA fragments are as described in Figure 2A; parental homologs “Mom” and “Dad”, the two CO-products, and DSBs. (ii) Darker exposure of the “DSB” region. B. Quantification of signals in the DSB region in A. (PDF) [file pgen.1003545.s002.pdf]

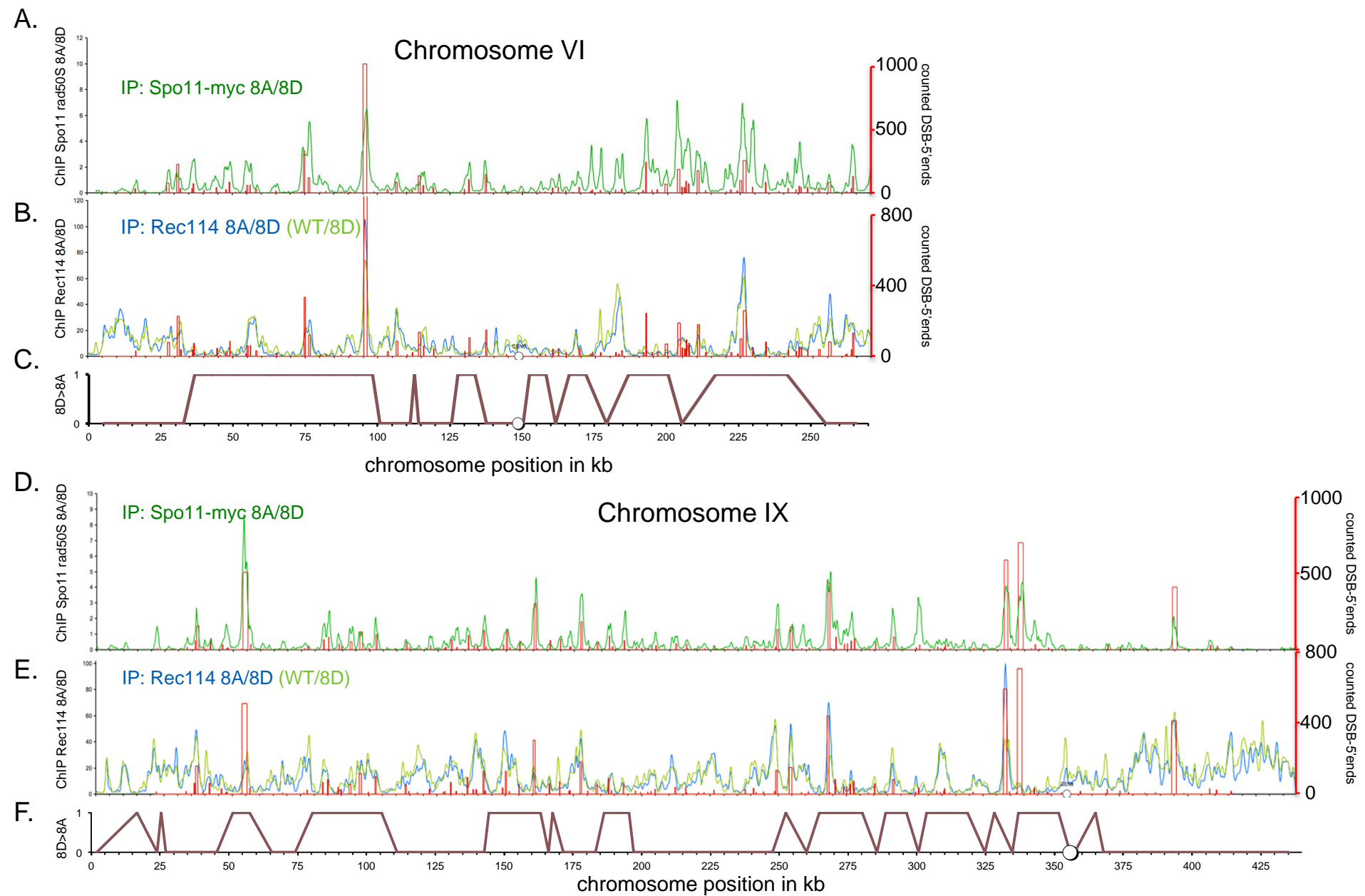

Supplement: Figure S3 — DSB sites match peaks of Spo11-myc and Rec1148A profiles. A. Spo11-myc profile of a rec114-8A rad50S strain normalized (divided) by Spo11-myc profile of a rec114-8D rad50S strain (green, “Spo11-8A/8D”). Red bars represent Spo11-oligo counts per hotspot cluster [7] Small chromosome VI is shown as an example to illustrate genome wide colocalization between Spo11-8A/8D peaks and DSBs. B. Rec114 profile of rec114-8A normalized (divided) by Rec114 profile of rec114-8D (blue, “Rec114 8A/8D”) and REC114 normalized by rec114-8D (bright green, “WT/8D”). Red bars represent Spo11-oligo counts per hotspot cluster [7]. Small chromosome VI is shown as an example to illustrate genome wide colocalization between peaks of Rec1148A/Rec1148D and Rec114/Rec1148D and DSBs. C. At axis sites defined by peaks of the axis protein Hop1 [17], “1” was plotted, if 8D/8A exceeded a certain threshold (0.5), while “0” was plotted otherwise. Both, groups of “1 s” and groups of 0 s” cluster together in the hot and cold DSB domains, respectively (50 axis sites). E., D., F. As in A., B., C. but on the larger chromosome IX. F. is built from 78 axis sites. (PDF) [file pgen.1003545.s003.pdf]

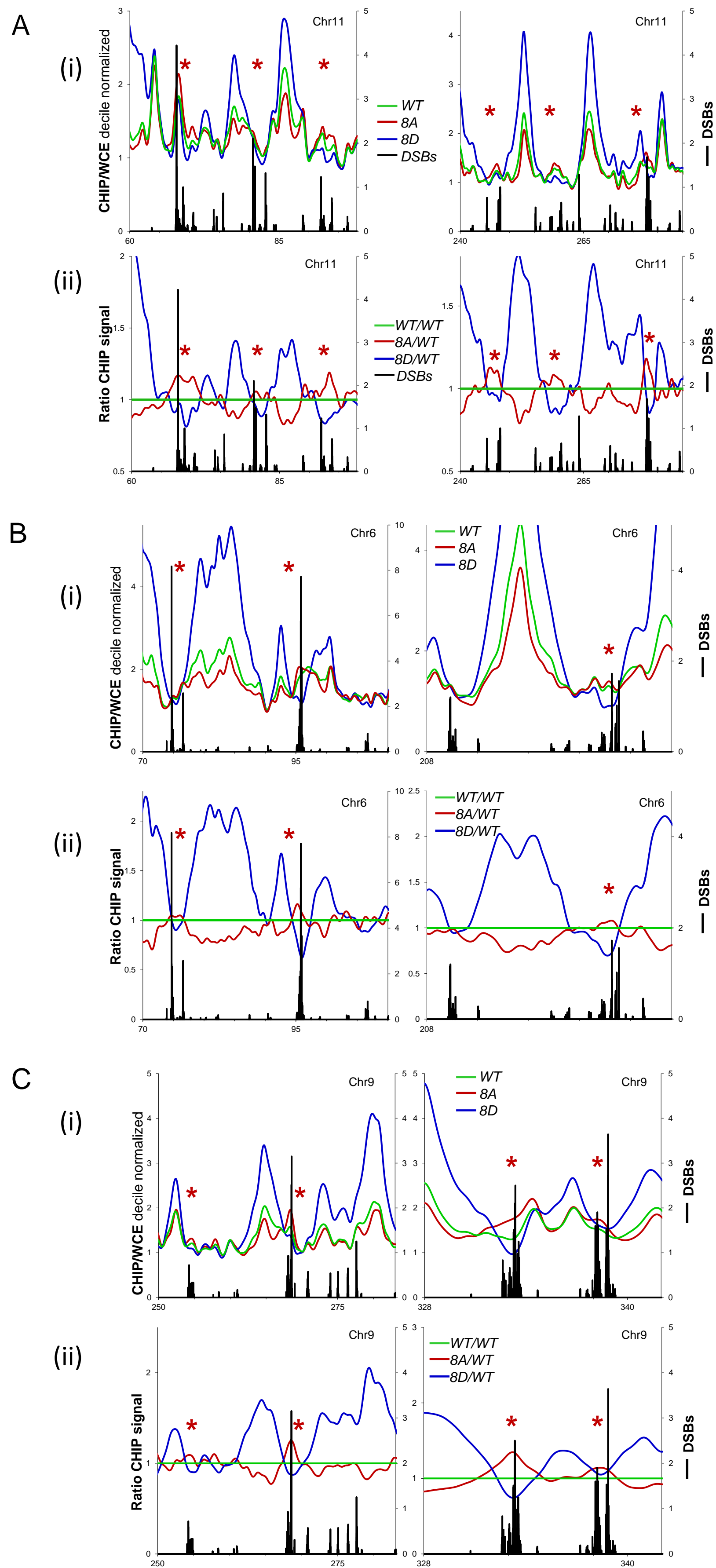

Supplement: Figure S5 — Additional examples of the mirror-like behavior of Rec1148A versus Rec1148A at DSB hotspots. Rec114 ChIPchip profiles of REC114 (green), rec114-8A (red), and rec114-8D (blue) are shown for selected regions in ChrVI (A), ChrIX (B), and ChrXI (C). * denotes strong DSB hotspots where Rec114 signal is highest in rec114-8A followed by REC114 and then by rec114-8D (upper panel). Ratio between signals from each mutant over wild type (lower panel) shows that majority of DSB hotspots are loci showing 8A>WT>8D quantitative relationship, while nearby axis shows 8D>WT>8A. For very weak hotspots, however, it is difficult to discern the 8A>WT>8D relationship as the positive effect of rec114-8A becomes insignificant compared to the negative effect of rec114-8D. (PDF) [file pgen.1003545.s005.pdf]

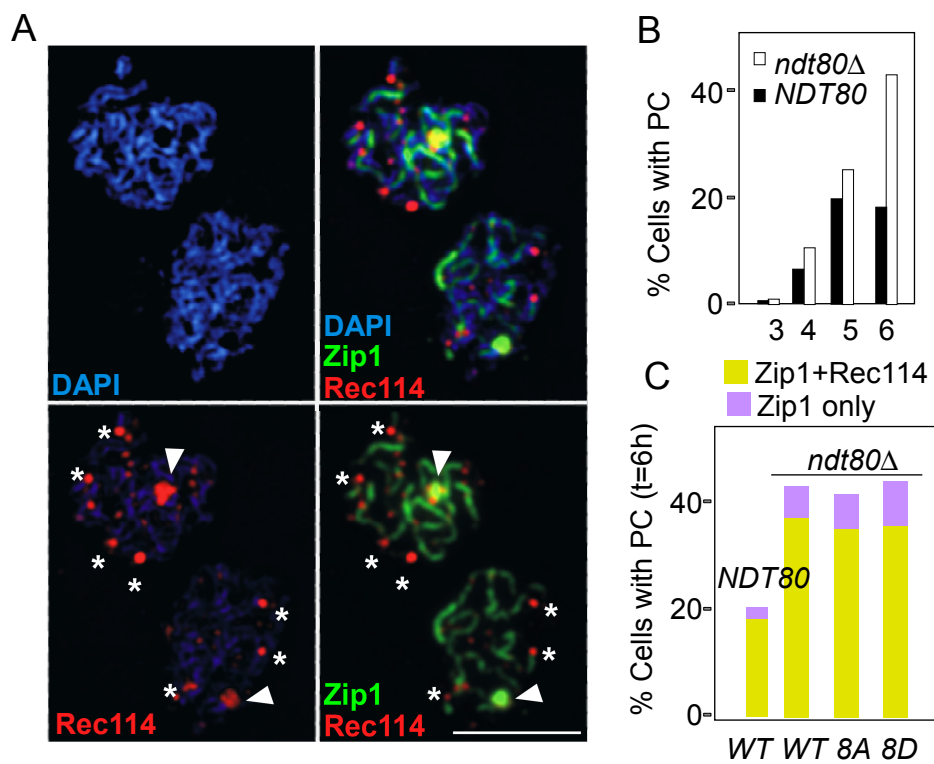

Supplement: Figure S6 — Rec114 foci persist in the ndt80 background. A. Representative images of REC114 ndt80Δ cells at t = 6 hours showing persistent Rec114 foci in Zip1 free regions or a PC (stars). B. Fraction of cells showing a polycomplex (PC) in REC114 NDT80 (black columns) or REC114 ndt80Δ (white columns) cells as a function of time. C. Fraction of cells showing a PC at t = 6 hours in the indicated strain background. Majority of PCs contained both Zip1 and Rec114 signals (yellow). (PDF) [file pgen.1003545.s006.pdf]
