# Supplementary material for: Budding Yeast ATM/ATR Control Meiotic Double-Strand Break (DSB) Levels by Down-Regulating Rec114, an Essential Component of the DSB-machinery
Source: PLoS Genet. 2013 Jun 27;9(6):e1003545. doi: 10.1371/journal.pgen.1003545 (PMC3694840; doi:10.1371/journal.pgen.1003545)

Carballo Figure S4

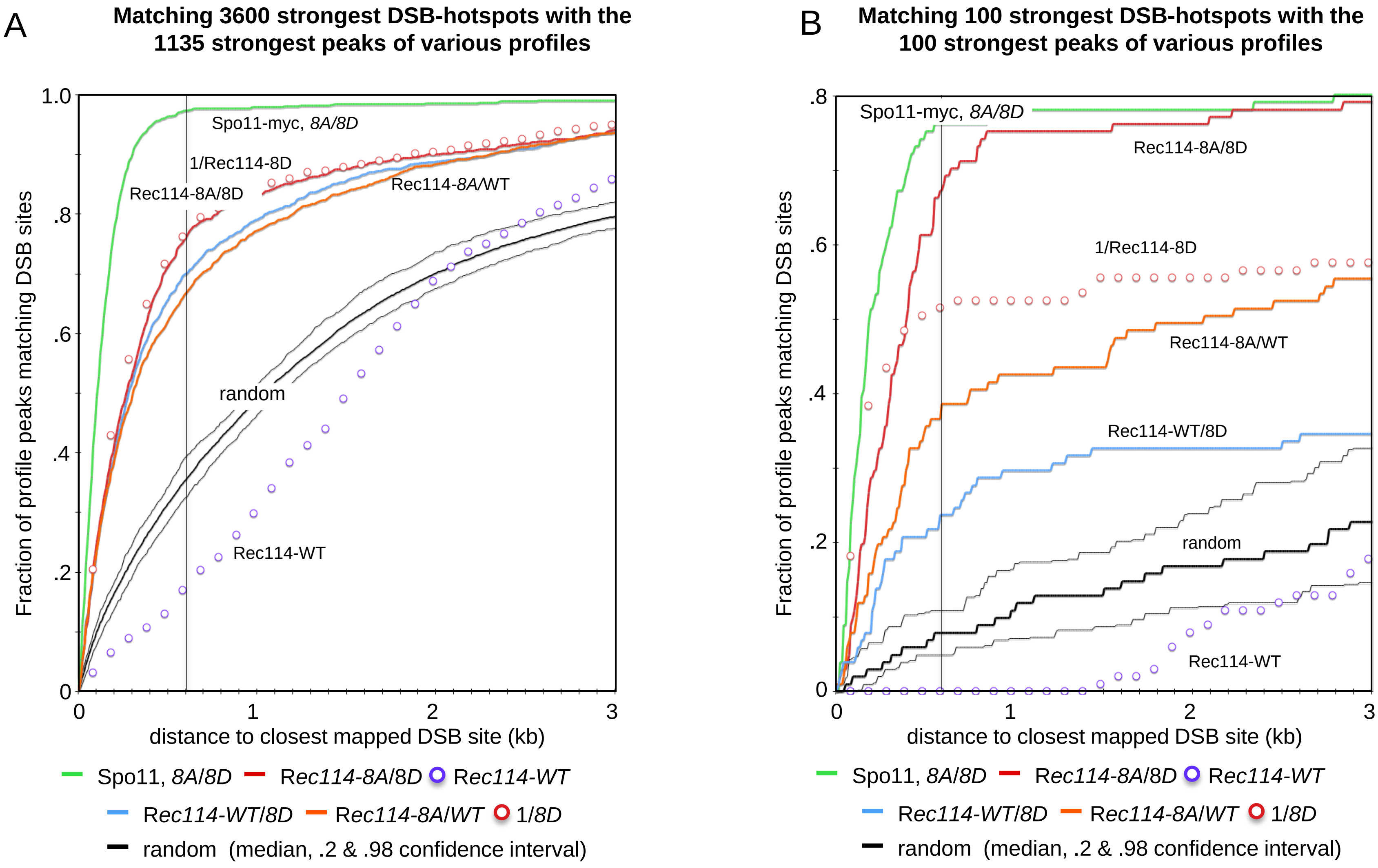

Supplement: Figure S4 — Genome wide correlation between DSB hotspots and peaks of Spo11-myc and Rec1148A profiles. A. The cumulative fraction of peaks of a specified profile is plotted against the distance from the nearest DSB cluster (in kb). Results of comparison between 3600 DSB sites [7] and the 1135 strongest peaks of various profiles are presented. B. Same as in A, but the comparison was between 100 strongest DSB hotspots and 100 strongest peaks of various profiles. (PDF) [file pgen.1003545.s004.pdf]
