# Supplementary material for: Budding Yeast ATM/ATR Control Meiotic Double-Strand Break (DSB) Levels by Down-Regulating Rec114, an Essential Component of the DSB-machinery
Source: PLoS Genet. 2013 Jun 27;9(6):e1003545. doi: 10.1371/journal.pgen.1003545 (PMC3694840; doi:10.1371/journal.pgen.1003545)
Supplement: Table S1 — S. cerevisiae strains used in this study. All strains are MATa/MATα diploids homozygous unless specified. For HIS4LEU2 hotspot recombination assay diploid strains JCY1193 (REC114), JCY1195 (rec114-8AQ) and JCY1197 (rec114-8DQ) were derived from NHY187 (Mata, ho::hisG, leu2, ura3, HIS4-LEU2NewBamH) and NHY285 (Matα, ho::hisG, leu2, ura3, his4X-LEU2NewBamH-URA3). (PDF) [file pgen.1003545.s007.pdf]

Table S1. *S. cerevisiae* strains used in this study

Strain	Genotype
NKY611	*ho::LYS2/ho::LYS2, ura3/ura3, lys2/lys2, leu2::hisG/leu2::hisG*
RCY1955	*ho::LYS2/ho::LYS2, ura3/ura3, lys2/lys2, leu2::hisG/leu2::hisG, REC114-8xMYC::URA3/REC114-8xMYC::URA3*
JCY919	As *RCY1955* but *rad24Δ::LEU2, tel1Δ::hphMX4*
JCY911	As *RCY1955* but *spo11-Y135F-URA3*
JCY922	As *RCY1955* but *dmc1Δ::KanMX4*
JCY216	As *RCY1955* but *rad50-K81I::URA3*
JCY452	*mec1Δ::LEU2, arg4::mec1-4-KanMX4, tel1Δ::hphMX4, sml1Δ::URA3*
RCY336	*Mat a ho::LYS2, lys2, ura3, leu2::hisG, rec114Δ::KanMX4*
RCY337	*Mat α ho::LYS2, lys2, ura3, leu2::hisG, rec114Δ::KanMX4*
RCY2613	*ho::LYS2/ ho::LYS2, lys2/lys2, ura3/ura3, leu2::hisG/leu2::hisG, rec114Δ::KanMX4/rec114Δ::KanMX4*
JCY948	As RCY2613 but *rec114Δ::REC114-13XMYC-hphMX4*
JCY979	As RCY2613 but *rec114Δ::rec114-S148A,T175A,S179A,S187A,S229A,T238A, S256A,S307A-13XMYC-hphMX4*
JCY1383	As RCY2613 but *rec114Δ::rec114-S148D,T175D,S179D,S187D,S229D,T238D, S256D,S307D-13XMYC-hphMX4*
FKY4583	As RCY2613 but *rec114Δ::REC114-hphMX4*
FKY4586	As RCY2613 but *rec114Δ::Δ::rec114-S148A,T175A,S179A,S187A,S229A, T238A,S256A,S307A-hphMX4*
FKY4589	As RCY2613 but *rec114Δ::Δ:: rec114-S148D,T175D,S179D,S187D,S229D, T238D,S256D,S307D-hphMX4*
JCY1511	As RCY2613 but *rec114Δ::REC114-hphMX4, ndt80Δ::LEU2*
JCY1515	As RCY2613 but *rec114Δ::rec114-8AQ-hphMX4, ndt80Δ::LEU2*
JCY1519	As RCY2613 but *rec114Δ::rec114-8DQ-hphMX4, ndt80Δ::LEU2*
JCY1034	As RCY2613 but *rec114Δ::REC114-hphMX4, sae2Δ::KanMX4*
JCY1037	As RCY2613 but *rec114Δ::rec114-8AQ-hphMX4, sae2Δ::KanMX4*
JCY1153	As RCY2613 but *rec114Δ::rec114-8DQ-hphMX4, sae2Δ::KanMX4*
RCY2691	As RCY2613 but *rec114Δ::REC114-hphMX4, dmc1Δ::KanMX4*
RCY2693	As RCY2613 but *rec114Δ::rec114-8AQ-hphMX4, dmc1Δ::KanMX4*

JCY1157	As RCY2613 but *rec114Δ::rec114-8AQ-hphMX4*, *dmc1Δ::KanMX4*
JCY1667	*ho::LYS2/ ho::LYS2, lys2/lys2, ura3/ura3, leu2::hisG/leu2::hisG, trp1/trp1, rad50S::URA3/rad50S::URA3, SPO11-MYC18::TRP1/SPO11-MYC18::TRP1, rec114Δ::REC114::hphMX4/ rec114Δ::REC114::hphMX4*
JCY1658	As JCY1667 but *rec114Δ::rec114-8AQ::hphMX4*
JCY1662	As JCY1667 but *rec114Δ::rec114-8DQ::hphMX4*
JCY1560	*Mat a ura3-1 trp1-28 leu2Δ0, lys2Δ0 his7 mob1::kanMX4 pep4::LEU2, GST-REC114-MOB1::TRP1*
JCY1553	As JCY1560 but *GST-rec114-8A-MOB1::TRP1*
RCY2123	*ho::hisG, ura3, leu2, MEC1-Myc18-LEU2*
JCY1207	*ho::LYS2/", lys2/", ura3/", leu2::hisG/", rec114::3xMYC-REC114-hphMX4/"*
JCY1222	As *JCY1207* but *rec114::3xMYC-rec114-8A-hphMX4/"*
JCY1210	As *JCY1207* but *rec114::3xMYC-rec114-8D-hphMX4/"*
JCY1269	*ho::LYS2/", lys2/", ura3/", leu2::hisG/", rec114D::REC114-hphMX4/", spo11-3HA-6HIS::KanMX4/"*
JCY1272	As *JCY1269* but *rec114D::rec114-8A-hphMX4/", spo11-3HA-6HIS::KanMX4/"*
JCY1273	As *JCY1269* but *rec114D::rec114-8D-hphMX4/", spo11-3HA-6HIS::KanMX4/"*
RCY2728	*ho::LYS2/", ura3/", lys2/", leu2::hisG/", his4B::LEU2/", spo11-D290A-HA3-His6::KanMX4/"*
JCY1159	As *RCY2728* but *rec114::rec114-8A::hphMX4/"*
JCY1161	As *RCY2728* but *rec114:: rec114-8D::hphMX4/"*
JCY998	*ho::LYS2/", lys2/", ura3/", leu2::hisG/", rec114::REC114-hphMX4/", pch2D::KanMX4/"*
RCY2688	As *JCY998* but *rec114::rec114-8A-hphMX4/"*
JCY1155	As *JCY998* but *rec114::rec114-8D::hphMX4/", pch2D::KanMX4/"*
