# Supplementary material for: Budding Yeast ATM/ATR Control Meiotic Double-Strand Break (DSB) Levels by Down-Regulating Rec114, an Essential Component of the DSB-machinery
Source: PLoS Genet. 2013 Jun 27;9(6):e1003545. doi: 10.1371/journal.pgen.1003545 (PMC3694840; doi:10.1371/journal.pgen.1003545)
Supplement: Text S1 — Supplementary methods. (DOCX) [file pgen.1003545.s008.docx]

**Text S1: SUPPLEMENTARY METHODS**

Carballo et al.

**Yeast strains and media**

Standard yeast manipulation procedures and growth media were utilized. All strains are of the SK1 background; relevant genotypes of the strains are listed in Table S1.

**Construction of *rec114* strains**

The myc13 tag from a *REC114-MYC13-HYGRO* plasmid (pNS2) was removed to generate pJC15, an integration plasmid without an epitope tag. Specific [S/T]Q to AQ or DQ mutations were introduced into either pNS2 or pJC15 utilizing the QuickChange Multi Site-Directed Mutagenesis kit (Stratagene). The entire open reading frame (ORF) of each allele was sequenced to ensure that the allele did not contain any incidental mutation(s). Each *rec114* allele was introduced into a *rec114Δ::KanMX4* haploid strain (RCY336/337), where the endogenous *REC114* gene was replaced by a kanamycin resistant gene. Transformants were identified based on their ability to grow on hygromycin plates but not on kanamycin. Southern blot and PCR analyses were performed on candidate colonies to confirm integration of a single copy of a specific *rec114-HygroMX4* allele at the endogenous locus, replacing the *rec114Δ::KanMX4* allele. Correct *rec114* haploid transformants of each allele were taken through standard yeast genetics manipulation to generate corresponding *rec114* homozygous diploid strains suitable for meiotic analyses.

**Generation of phospho-specific Rec114 antibodies**

Three of the eight S/T[Q] consensus sites in Rec114, T175, S187 and S256, were selected for generation of phospho-specific antibodies. T175 and S187 were chosen based on the fact that replacing these residues with a non-phosphorylatable alanine (A) confers haploinsufficiency and synthetic interaction with *spo11* hypomorphic alleles (Table 1). S256 was chosen because it was one of the six residues within Rec114 that were predicted to be the most likely ATM/ATR phosphorylation sites (GPS2.1 software [[1](#_ENREF_1)]). Specificity of each phospho-specific antibody was confirmed by Western blot analysis of *rec114* strains, each expressing a *rec114* allele missing a specific phosphorylation site(s).

**Synchronous meiotic time course**

Induction of synchronous meiosis is carried out according to the established protocols [[2](#_ENREF_2),[3](#_ENREF_3)]. All pre-growth and meiotic time courses were carried out at 30^o^C except for *mec1-4^ts^ tel1Δ sml1Δ* meiosis, where the culture was kept at 23^o^C and shifted to 30^o^C 2 hours after transferring into sporulation medium (SPM).

**Protein purification and manipulation methods**

*GST-REC114* and *GST-rec114-8A* plasmid-construction and protein expression were carried out as described [[4](#_ENREF_4)]. To purify Mec1-myc18 from yeast cells, 500 ml of logarithmically growing cell cultures were subjected to 1 hour incubation with 0.1% methyl methanesulfonate (MMS) followed by Immunnoprecipitation using Goat anti-myc-agarose antibodies (AbCam). Mec1-myc immunoprecipitates were mixed with reaction cocktail containing kinase buffer, cold ATP, and either GST-Rec114 or GST-Rec114^8A^. The mixtures were incubated at 30^o^C for 25 minutes and subjected to electrophoresis on SDS gels. Gels were transferred onto a nitrocellulose membrane and subjected to Western blot analyses using anti-Rec114 or phospho-specific antibodies.

**Western blot analysis.**

Whole-cell extracts (WCE) were prepared from cell suspensions in 20% trichloroacetic acid (TCA) by agitation with glass beads. Precipitated proteins were solubilized in SDS-PAGE sample buffer, and appropriate dilutions were analyzed by SDS-PAGE and Western blotting. Antibodies for Western blotting were mouse monoclonal anti-myc (1:1000, AbCam), rabbit polyclonal anti-Rec114 (1:1000), anti-Phospho-Rec114-S187, anti-Phospho-Rec114- T175, anti-Phospho-Rec114- S265 (1:1000, Cambridge Research Biomedicals), goat anti-mouse IgG conjugated to horseradish peroxidase (1:10,000; Sigma-Aldrich), and donkey anti-rat IgG conjugated to horseradish peroxidase (1:10,000; Sigma-Aldrich)

**Southern blot analysis**

Southern blot analysis following Pulse Field Gel electrophoresis (PFGE) using DNA prepared in agarose plugs or standard agarose gel electrophoresis were performed as described [[5](#_ENREF_5)]. Exception was that the PFGE gels shown in Figure 2G and Figure S1A were run with the following modifications: initial switch time; 15 sec – final switch time; 32.5 sec, in order to better separate large chromosomes. For quantifying the level of DSBs, only the signals associated with breaks proximal to the probe was utilized to maximize the detection of chromosomes that acquired more than one break (see [[6](#_ENREF_6)] for discussion).

**Chromatin Immunoprecipitation on CHIP (ChIPchip) and quantitative PCR (qPCR)**

Rec114 and Spo11-myc chromatin immunoprecipitation (ChIP), quantitative PCR (qPCR), and microarrays hybridization/analysis were performed as described [[3](#_ENREF_3)].

**Cytological Methods**

Surface spread meiotic chromosomes were prepared as described [[7](#_ENREF_7)]. Staining was performed as described [[7](#_ENREF_7)] with the following primary antibodies: rabbit polyclonal anti-Rec1141 (1: 100, F. Klein, MFPL), mouse monoclonal anti-HA (12CAS, 1:100, S. Ley, NIMR), mouse monoclonal anti-MYC (9E10, 1:100, S. Ley, NIMR goat polyclonal anti-Zip1 (1:50, SantaCruz Biotechnology). Secondary antibodies (Invitrogen) were used at a 1:500 dilution: chicken anti-mouse Alexa-488, anti-goat Alexa-488, chicken anti-rabbit Alexa-594. Chromosomal DNA was stained with 1ug/ml 4,6-diamino-2-phenylimide (DAPI). Images were recorded and analyzed using a Deltavision (DV3) workstation from Applied Precision Inc. with a Photometrics CoolSnap HQ (10-20MHz) air cooled CCD camera and controlled by Softworx image acquisition and deconvolution software.

**REFERENCES CITED IN SUPPLEMEMTARY INFORMATION**

1. Xue Y, Ren J, Gao X, Jin C, Wen L, et al. (2008) GPS 2.0, a tool to predict kinase-specific phosphorylation sites in hierarchy. Mol Cell Proteomics 7: 1598-1608.

2. Padmore R, Cao L, Kleckner N (1991) Temporal comparison of recombination and synaptonemal complex formation during meiosis in *S. cerevisiae*. Cell 66: 1239-1256.

3. Panizza S, Mendoza MA, Berlinger M, Huang L, Nicolas A, et al. (2011) Spo11-accessory proteins link double-strand break sites to the chromosome axis in early meiotic recombination. Cell 146: 372-383.

4. Geymonat M, Spanos A, Jensen S, Sedgwick SG (2010) Phosphorylation of Lte1 by Cdk prevents polarized growth during mitotic arrest in *S. cerevisiae*. J Cell Biol 191: 1097-1112.

5. Murakami H, Borde V, Nicolas A, Keeney S (2009) Gel electrophoresis assays for analyzing DNA double-strand breaks in *Saccharomyces cerevisiae* at various spatial resolutions. Methods Mol Biol 557: 117-142.

6. Borde V, Goldman AS, Lichten M (2000) Direct coupling between meiotic DNA replication and recombination initiation. Science 290: 806-809.

7. Carballo JA, Johnson AL, Sedgwick SG, Cha RS (2008) Phosphorylation of the axial element protein Hop1 by Mec1/Tel1 ensures meiotic interhomolog recombination. Cell 132: 758-770.
